# Engineered bio-functional material-based nerve guide conduits for optic nerve regeneration: a view from the cellular perspective, challenges and the future outlook

**DOI:** 10.1093/rb/rbae133

**Published:** 2024-11-22

**Authors:** Enoch Obeng, Baoguo Shen, Wei Wang, Zhenyuan Xie, Wenyi Zhang, Zhixing Li, Qinqin Yao, Wencan Wu

**Affiliations:** State Key Laboratory of Ophthalmology, Optometry and Vision Science, Wenzhou Medical University, Wenzhou, Zhejiang 325027, China; The Eye Hospital, School of Ophthalmology & Optometry, Wenzhou Medical University, Wenzhou 325027, China; State Key Laboratory of Ophthalmology, Optometry and Vision Science, Wenzhou Medical University, Wenzhou, Zhejiang 325027, China; The Eye Hospital, School of Ophthalmology & Optometry, Wenzhou Medical University, Wenzhou 325027, China; State Key Laboratory of Ophthalmology, Optometry and Vision Science, Wenzhou Medical University, Wenzhou, Zhejiang 325027, China; The Eye Hospital, School of Ophthalmology & Optometry, Wenzhou Medical University, Wenzhou 325027, China; State Key Laboratory of Ophthalmology, Optometry and Vision Science, Wenzhou Medical University, Wenzhou, Zhejiang 325027, China; The Eye Hospital, School of Ophthalmology & Optometry, Wenzhou Medical University, Wenzhou 325027, China; State Key Laboratory of Ophthalmology, Optometry and Vision Science, Wenzhou Medical University, Wenzhou, Zhejiang 325027, China; The Eye Hospital, School of Ophthalmology & Optometry, Wenzhou Medical University, Wenzhou 325027, China; State Key Laboratory of Ophthalmology, Optometry and Vision Science, Wenzhou Medical University, Wenzhou, Zhejiang 325027, China; The Eye Hospital, School of Ophthalmology & Optometry, Wenzhou Medical University, Wenzhou 325027, China; State Key Laboratory of Ophthalmology, Optometry and Vision Science, Wenzhou Medical University, Wenzhou, Zhejiang 325027, China; The Eye Hospital, School of Ophthalmology & Optometry, Wenzhou Medical University, Wenzhou 325027, China; State Key Laboratory of Ophthalmology, Optometry and Vision Science, Wenzhou Medical University, Wenzhou, Zhejiang 325027, China; The Eye Hospital, School of Ophthalmology & Optometry, Wenzhou Medical University, Wenzhou 325027, China; Oujiang Laboratory (Zhejiang Lab for Regenerative Medicine, Vision, and Brain Health), Wenzhou, Zhejiang 325000, China

**Keywords:** biomaterials, nerve guide conduits, optic neuropathy, optic nerve crush, regeneration

## Abstract

Nerve injuries can be tantamount to severe impairment, standard treatment such as the use of autograft or surgery comes with complications and confers a shortened relief. The mechanism relevant to the regeneration of the optic nerve seems yet to be fully uncovered. The prevailing rate of vision loss as a result of direct or indirect insult on the optic nerve is alarming. Currently, the use of nerve guide conduits (NGC) to some extent has proven reliable especially in rodents and among the peripheral nervous system, a promising ground for regeneration and functional recovery, however in the optic nerve, this NGC function seems quite unfamous. The insufficient NGC application and the unabridged regeneration of the optic nerve could be a result of the limited information on cellular and molecular activities. This review seeks to tackle two major factors (i) the cellular and molecular activity involved in traumatic optic neuropathy and (ii) the NGC application for the optic nerve regeneration. The understanding of cellular and molecular concepts encompassed, ocular inflammation, extrinsic signaling and intrinsic signaling for axon growth, mobile zinc role, Ca^2+^ factor associated with the optic nerve, alternative therapies from nanotechnology based on the molecular information and finally the nanotechnological outlook encompassing applicable biomaterials and the use of NGC for regeneration. The challenges and future outlook regarding optic nerve regenerations are also discussed. Upon the many approaches used, the comprehensive role of the cellular and molecular mechanism may set grounds for the efficient application of the NGC for optic nerve regeneration.

## Graphical Abstract

**Figure:**
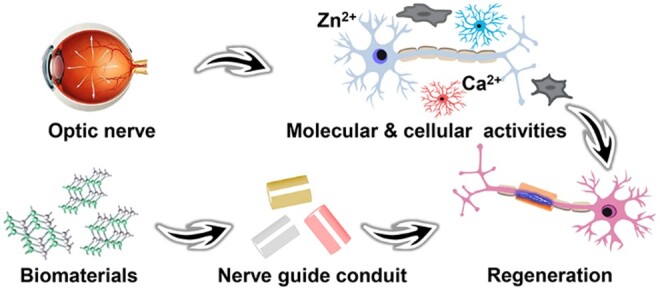

## Introduction

We still live in an era where the optic nerve is not able to regenerate, with no well-known measure available to completely repair an injured optic nerve. An attempt by a number of groups still gives hope that the future holds a mechanism to deal with the pending situation and over time it will be unraveled. The anatomical basis for sight recovery in an injured nerve is always premised on the reconnection between the regenerated RGC axons and the brain's visual nuclei. The optic nerve relays visual information from the retina to the brain, consisting of about over 1 million axons. These axons of the RGC span the length of the nerve fiber layer (NFL) merging as an axonal bundle. The NFL goes beyond the optic disc and is ensheathed by myelin from the oligodendrocyte. Visual information happens to be relayed to the visual cortex by the neurons in the lateral geniculate nucleus. Meanwhile, other visual-associated functions such as orienting, movement and circadian rhythm are undertaken by extra-nuclear pathways. However, the optic nerve in this state can still become a victim of impact or trauma associated with either side of the connection (the frontal or posterior part of the head) with millions of incidents and cases of blindness happening every day around the globe. Among some disorders involving the optic nerve are glaucoma, intra-ocular pressure-related disease, RGC and oligodendrocyte death, optic nerve atrophy and ischemic optic neuropathy (inflammation or poor circulation among the blood supply vessels of the optic nerve). Over time biomaterials have proved to be effective for biomedical usage and have been promising for therapeutic function and regenerative purposes. Since the discovery of four components of glass materials in 1969, materials that bonded living tissues have paved the way for their usage in dental and orthopedics [1–4].

Also, earlier research focused on the use of bio-ceramic and bioactive glasses for the regeneration of soft tissues, where cell modification and remodeling were accomplished via natural metabolism, a typical example is the calcium phosphates (CaPs). Next, hydroxyapatite (HA) which resembles an inorganic tissue was employed for the regeneration of hard tissues, rejuvenation and regeneration of the skin and acted as a skin barrier, shielding the skin from excessive water loss and other environmental effects [5–8]. Later advances in research led to the discovery of resorbable and biodegradable biomaterials. The extensive use of resorbable, bioinert and bioactive implants over a period of time gained ground although with challenges such as failure rate of implants and revision surgery. Today it can be said that biomaterials have become a lynchpin in the regeneration of tissue that has undergone traumatic or pathological lesions. In light of features such as composition, texture and mechanical performance, allowing for preferential differentiation of stem cells, tissue reformation, regeneration or remodeling [9–12]. Despite efforts and the prevalence of drugs of different origins, the overall outcome for optic nerve recovery remains unsatisfactory. These approaches encompass the use of pharmacological products, enhancing factors, topical injection, immune system modulators, etc. Along with this, is the unsatisfactory outcomes obtained from the treatment of individuals with related secondary complications arising from microsurgical procedures.

Steadfastly, research has also ceased not to give up on the understanding of the regeneration mechanism, and means to improve or recover the optic nerve. For some decades, research on the use of nerve growth factor (NGF) pointed out the improvement of nerve regeneration by topical agents. However, concerns with this conclusion are whether the approach was suitable for a severe and extended form of injury. All these efforts and their accompanying limitations point out the fact that the wholistic information regarding the cellular and molecular mechanism involving the injury cascade process, the degeneration and regeneration of the optic nerve is not clearly understood and has not been unraveled [13–16]. Regardless of the advances in this field, no review has thrown light on the cellular, molecular mechanism and NGC application. This review seeks to bridge this gap for the successful employment of the NGC for optic nerve regeneration. Hence, we provide a comprehensive review of ocular inflammation, ciliary neurotrophic factors (CNTF) gene therapies’ role in regeneration, microglial activation under optic nerve injury, the extrinsic and intrinsic factors, neurotrophic role in axon regeneration, mobile zinc role and the Ca^2+^ ion factor in injured optic nerve. The need for an alternative nanotechnological therapy based on the molecular information, a discussion on biomaterials for NGC fabrication and comprehensive information regarding NGC for optic nerve regeneration. Finally, the challenges, future outlook and conclusion. To this end, we aim to lay grounds for the extensive application of NGCs for the regeneration and recovery of the optic nerve.

## Ocular inflammation

Induction of sterile inflammation yields no defect but rather augments the RGC survival and axon regeneration. Similarly, the infiltration of neutrophils and macrophages is evident in lens injury where they play a crucial role in the RGC survival via the regulation of regeneration and axon growth. This means induction of such factors for RGC survival could be mimicked by Zymosan injection (intravitreal) which can further activate the Toll-like receptor 2 (TLRS2) and dectin-1, a pattern recognition receptor [17–22], under this state receptors of pro-inflammatory chemokines are blocked via the suppression of Zymosan activity. This raises the subject that neuroprotection and axon regeneration could be triggered by sterile inflammation. Under ocular inflammation, Oncomodulin (Ocm), 12 kDa Ca^2+^ binding neutrophile derived protein are released into the vitreous and subsequently the inner retina, the Ocm then binds to the RGC surface receptors via a cAMP-dependent manner [23–26]. Subsequently, the activity of Ocm is backed by stromal cell-derived factor 1 (SDF1) released by infiltrative macrophages under ocular inflammation. Reports indicate the enhancement of the effect of the Ocm, by SDFI stimulation in cell culture proved to regulate the axon outgrowth of the RGC and even the deletion of SDF1 in myeloid cells or CXCR4, as the primary SDF1 receptor is capable of suppressing the Zymosan-induced axon regeneration and RGC survival [26, 27]. Interestingly, with the exception of the αRGC, the SDF1 induces regeneration for other subtypes that respond to the deletion of phosphatase and tensin homolog (PTEN). Henceforward, the combinatorial role of the SDF1 under intraocular inflammation, Ocm/cAMP elevation and Pten deletion, in the axon regeneration and the survival of RGC. Studies indicate that the blockade of the Ocm function closely eliminates regeneration assumed to be induced by sterile inflammation. Meanwhile, exogenous SDF1 in combination with Ocm/cAMP enhances RGC survival via mimicking intraocular inflammation [20, 23, 28–30].

### Microglial activation under optic nerve injury

The mammalian retina has three forms of glial cells, (i) Müller cells form the largest portion of the total glial (∼80–90%) and are highly specialized microglial cells (ii) retinal astrocytes are found in the NFL of the retina and (iii) retinal microglia are found in the NFL and inner plexiform layer (IPL), forms about ∼5–20% of the total glial and are involved in the maintenance of the retinal homeostasis. The microglia modulate the retinal synapse, surveillance and immunocompetence of the cells in the neural environment [31–40]. Their possession of unique morphological and immunohistochemical features separate them from the other cells in the retina and are easily differentiated through the employment of some markers such as CD11b, CD68, F4/80 or ionized calcium-binding adapter molecule 1 (IBA1). Studies show that non-homeostatic microglia can be associated with photoreceptor degeneration and even neurodegenerative disorders [41–47]. Following an optic nerve crush (ONC), microglia become activated, in the process macrophages and other round cell bodies are recruited to infiltrate the site of injury. These resident microglia and infiltrating macrophages then become engaged in the degradation and expelling of myelin debris (Wallerian degeneration) [48–53]. Unlike the peripheral nervous system (PNS) where infiltrating macrophages clear up the myelin debris, allowing the growth permissive environment for the regeneration of the motor axon and subsequent recovery of the motor function. In the CNS, this does not just happen rapidly as indicated in the PNS.

**Figure 1.**
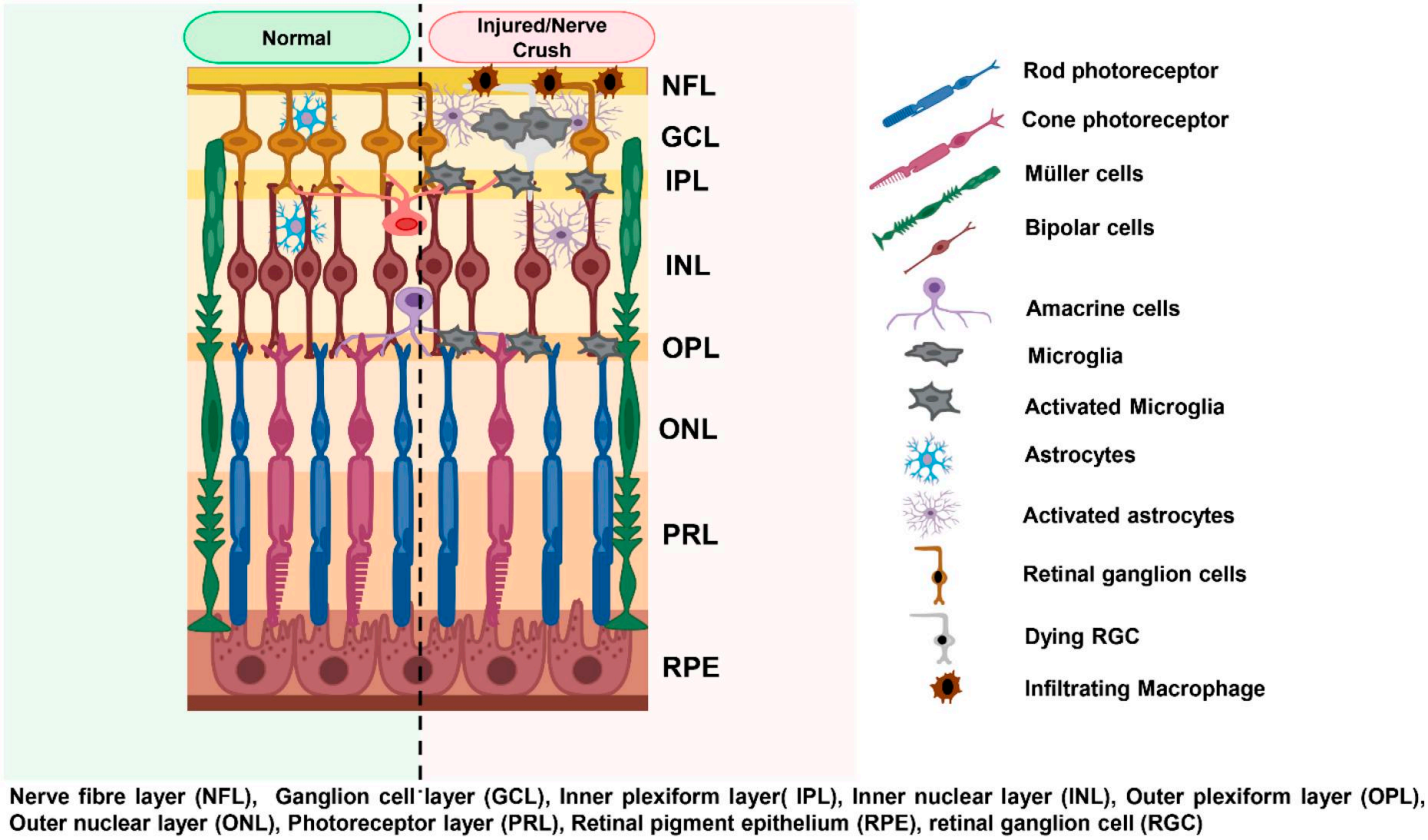
Schematic representation of the inflammatory response following ONC.

However, the oligodendrocytes responsible for the clearing of myelin debris undergo apoptosis, and in the process, its precursor fails to proliferate [51, 54–58]. Also, the fact that the resident microglia could have taken up this task and accomplished it within a short time, regardless, the assumption of infiltrating macrophage and resident microglia are not immediately observed following ONC. Consequently, with time the activated retinal microglia and the macrophage infiltration take place. Despondently, at this stage, the clearance of myelin debris by the resident microglia and the activity of macrophage becomes somewhat ineffective (Figure 1) [59–63]. Interestingly the elevated levels of retina microglia may be accompanied by increasing neurotoxic and pro-inflammatory cytokine resulting in neuronal loss and axon degeneration. This also occurs under the influence of chronic glial scarring, where inflammation, reactive gliosis and glial scar formation characterize the injured CNS. The reactive astrocytes in their facilitation of the damaged tissue repair through the formation of glial scar limit the dissemination of inflammation. Through the mediation of STAT3 (master regulators), there is an increase in rigidity, this rigidity further allows the formation of the glial scar [31, 64]. Subsequently, the continual scarring formation exceeding the acute phase injury blocks the axon regeneration and recovery [65–69]. The formation of these dense network encapsulation acts as a physical and chemical barrier preventing the intrusion of inflammatory agents and the blockade of the axon growth into the lesion location [64, 70–74]. Further, the reactive astrocyte releases chondroitin sulfate proteoglycans (CSPG) at the injury site, meanwhile regenerating axons in an attempt to penetrate the glial scar produces a dystrophic swollen retraction bulb (growth cone collapse). Axonal growth at this stage is halted limiting it to the glial scar. Thereupon, the chronic glial scarring induces and paves the way for resident microglia and infiltrating macrophage to be accompanied by neurotoxic and pro-inflammatory cytokines. An increasing number of infiltrating macrophages at the site of injury can also override the reactive astrocyte activity and glial scar, allowing for the clearance of myelin debris and also the regeneration of axon across the glial scar (a switch from the neurotoxic M1-like phenotype to neuroprotective M2-like phenotype). At this point, infiltrating macrophage and resident microglia do not hinder the axon growth but enhance the debris clearance and axon regeneration [75–83].

#### Activated microglia, RGC survival and axon regeneration

The induction of neuronal apoptosis following ONC can trigger the activation of resident retinal microglia and macrophage infiltration in the proximal nerve stump and within the retinae. However, during prolonged activation of resident microglia, M1 makers (IL-Iβ, IL-6 and TNFα) are elevated whiles M2 makers (CD206, Ym1 and Arg 1) are significantly suppressed which triggers neuronal apoptosis and the substantial worsening of other secondary tissue damage. Also, the upregulation of inducible nitric oxide synthase (iNOS) in the microglia has the potential to cause RGC death [84–89]. The reactive microglia through the expression of some proteins such as TNFα, IL-1α and C1q, induce the polarization of the astrocyte to an inflammatory state (A1 astrocyte). A1 astrocyte modulates synapse degradation and cell death of other neurons and oligodendrocytes via a long chain saturated lipid (APOE and APOJ containing lipoparticles) [90–99]. While genetic ablation of complement proteins such as C1q, C3 and CR3 receptors impedes the axon regeneration in adult mice and reduces the myelin basic protein (MBP) clearance at a lesion site [20–22, 24, 100–103]. These complement proteins and CR3 receptor role have been linked to the resident microglia since the depletion of the resident retinal microglia by PLX5622, CX3CR1 antagonist result in the reduced expression of C1q, C3 and CR3 receptor following ONC. The PLX5622 together with its structural homolog PLX339 can deplete microglia in binding with the colony-stimulating factor 1 receptor (CSF1R) in adult CNS [104–108]. Reports indicate that the elimination of microglia resulted in less expression of Aif1 and C1qa however CR3+ monocyte increased at the injury site [100, 109].

**Figure 2.**
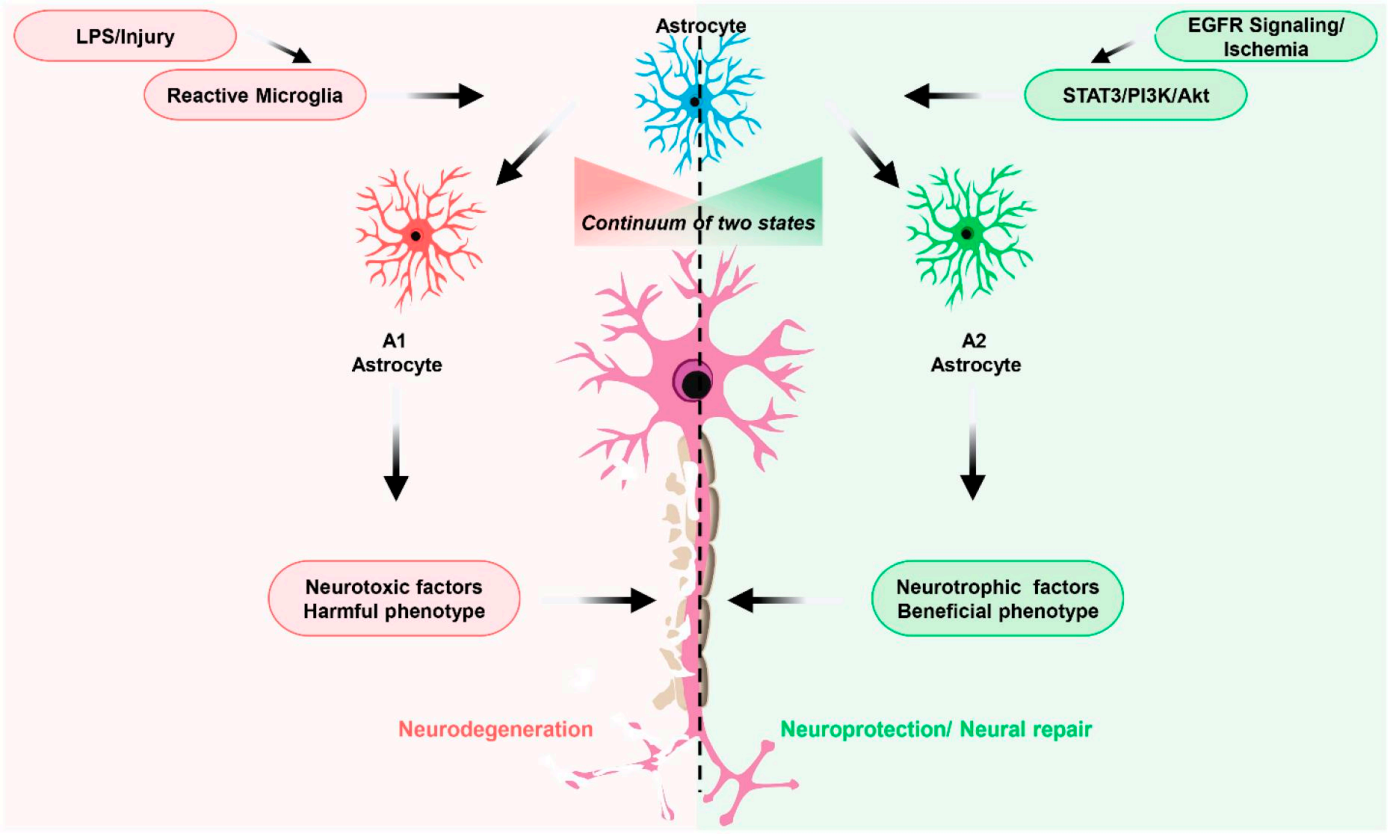
Schematic representation of the yield of A1 and A2 astrocytes via injury and ischemia respectively.

Accumulating evidence suggests that astrocytes and Müller cells become activated under ONC, where Müller cells are known to be activated by the elevation of intraocular glutamate which triggers the release of adenosine triphosphate (ATP) [110–113]. The Müller cells being the largest constitute the glial cells stem from the retinal epithelium and span across the entire retinal mass. The Müller cells permit metabolic exchange among the RGC and retinal vasculature. Also, the astrocyte is involved in the formation of the blood retinal barrier thus playing a crucial role in the retinal vascular system and is essential for axonal regeneration and neuronal survival [31, 114–116]. The stabilization of a tight junction among the endothelial cells paves the way for the modulation of the blood–retina barrier integrity, essential nutrient supply (lactate and amino acids), glucose metabolism, retinal neurotransmitter and blood flow, which happens to be some major roles played by the astrocyte and Müller cells [31, 70, 117, 118]. As the astrocytes and Müller cells engage in the release of CNTFs and brain-derived neurotrophic factors (BNDF), also the Müller cells can engage in the production of neurotrophic factors such as glial cell line-derived neurotrophic factors (GDNF) and NGFs [119–123]. Studies show the Müller cells are able to release CNTF following lens injury and thus an indication of their cooperation with the astrocytes for the promotion of the regeneration of RGC [20, 124, 125]. As mentioned earlier, the astrocytes may function under two reactive states since under LPS-induced neuroinflammation, the astrocyte could be linked to the classical complement cascade expression, yielding synapse loss and neurodegeneration (A1 phenotypes). However, under ischemic injury, the expression of neurotrophic factors and cytokines for neural repair is accomplished by the activated astrocyte (A2 phenotype). Neurotoxic A1 phenotype and neuroprotective A2 phenotype explain the distinction existing between the reason the astrocyte could be involved in the neuronal loss or survival (Figure 2) [96, 126–133]. Under ONC, an A1 astrocyte is formed and hence targeting complement cascade, IL-1α and TNFα could act as a blockade for this A1 phenotype induction [97, 134–138].

#### Effect of the astrocyte via EGFR expression

Activation of the intrinsic RGC’s growth ability seems to be one major focus under the injury of the optic nerve. The epidermal growth factor (EGF) signaling modulates a broad range of development processes (differentiation, proliferation, migration, etc.) with their signal occurring via EGF receptors and other homologous receptors (ErbB2, ErbB3 and ErbB4). They pave the way for the signaling of protein complexes that are involved in the PI3K/Akt and STAT3 pathway following dimerization and phosphorylation of their tyrosine residue [139–141]. Although there hadn’t been enough studies emphasizing the EGFR signaling role concerning axon regeneration and RGC survival, however, an increase of EGFR expression in adult retinas indicates their peculiar role following injury. Also, reports indicate the suppression of the retinal EGFR following the retina maturation caused the loss of their mitogenic response. The astrocyte has been used to exert its protective effect via the EGFR signaling since the EGFR signaling mediates the upregulation of complement C3 in astrocytes, hence the protection of the RGC from high intraocular pressure [97, 142–144]. The presence of complement protein C3 still raises the question of the A1 phenotype being neurotoxic or neuroprotective but studies suggest the continuum existence of the astrocyte having a heterogeneous population (both A1 and A2). However, the impact of the EGFR on axon regeneration and RGC survival, their association with the astrocyte and the phenotype involved (A1 and A2) remain to be fully elucidated and understood (Figure 2) [31, 97, 133, 143, 145].

#### Activated microglia and remyelination

Myelination is essential for shielding the regenerating axons from degenerating, allowing the long-distance axon regeneration towards a point for reinnervation (distal brain target). Unfortunately, unmyelinated regenerating axon failure to innervate their target immediately assumes degeneration. Although much emphasis is placed on the long-distance regeneration of the axon, remyelination seems to be under-focused, meanwhile, studies show that poor myelination results in poor recovery of visual associated behaviors following ONC. Remyelination in itself happens not to occur promptly over the entire length or distance. Nevertheless, myelination occurs under the rapid proliferation of oligodendrocyte percussor cells (OPCs) to permit proper nerve conduction, following differentiation into matured oligodendrocytes and ensheathing of the regenerating axon [28, 146–148]. Studies show the M1 polarized microglia and macrophage characterized the early period of the OPCs induction and are involved in the release of varying genes for the clearance of myelin debris (C1q complement protein and CX3CR1). Although, even before remyelination set in they are expelled via necroptosis. Nonetheless, under the M2-like phenotype, the polarization of the microglia and macrophages promoted remyelination. It’s worth noting that, blocking associated distinct markers (arginase 1, CD206 and IGF-1) could still impede the M2 polarization of microglia and macrophage, hence the connection between the differentiation of oligodendrocyte and microglia polarization for remyelination [53, 149, 150]. This concludes on the fact that the microglia are involved in remyelination. Also depleting the activated microglia can inhibit the proliferation of OPCs in ONC (first 2 weeks), and adoption of M1 phenotype by microglia could lead to the expression of high level iNOS, which does not have a good prospect for remyelination [86, 148].

#### Zymosan with other regulatory factors for regeneration

An indication that there may be crosstalk between resident microglia, the astrocyte and Müller cells in connection with the infiltration of myeloid cells in an injured retina, is how the intravitreal injection of Zymosan results in the release of Müller cell-derived growth factors and axon regeneration, following the activation of retinal astrocyte and Müller cells. Meanwhile, intra-optic injection of Zymosan does not yield such an effect [21, 22, 151, 152]. Also, the sole treatment with Zymosan has a negligible effect on axon regeneration and growth over a long distance. However, the combinatorial treatment (Zymosan + Pten deletion, lipid and protein phosphatase involved in the suppression of signaling for the RGC’s axon regeneration via PI3K/Akt pathway) as mentioned earlier induced axon regeneration via mammalian target of rapamycin (mTOR) pathway an essential PI3 kinase downstream target and a central cell growth regulator. Accumulating evidence suggests the prevention of mTOR inhibition and the upregulation of a proximate upstream regulator (cRheb1) can allow for the optic nerve regeneration via the synergistic activity of cRheb and RGC, permitting the regeneration of axon to the central targeted area [69, 153, 154]. Also, studies show that Zymosan-induced axon regeneration is known to be mediated by β-glucan/dectin-1 (phagocytic receptor) signaling cascade, and genetic ablation of dectin-1 blocks the Zymosan induced axonal regeneration [155–157]. Next, another factor is the deletion of suppressor of cytokine signaling-3 (SOCS3) from the RGC, which could contribute to optic nerve regeneration, a repressor of Jak-STAT signaling, CNTF and its related factors. Studies indicate that the deletion of CNTF and its related factors (LiF) or STAT3 hampered the Zymosan effect or even the growth promoting effect associated with the lens injury [158–161]. Hence the deletion of SOCS3 and Pten seeks to fully allow the functioning of the CNTF, numerous axon regeneration spanning the full length of the optic nerve [162–166].

Interestingly, recognition of the role of transcription factor also holds the possible enlightenment on the conditions surrounding the optic nerve regeneration. The Klf4 a member of the Krüppel-like family (KLF), with other transcription factor (Oct4 and Sox2) has an effect on DNA methylation and transcriptome in the RGC when overexpressed following an optic nerve injury. The Klf4 and Klf9 (more pronounced) deletion can enhance axon regeneration following optic nerve injury [167–169]. Next, the leucine zipper bearing kinase (LZK), referred to as the MAP3K13, a mitogen activated protein kinase kinase kinase with about 90% identical amino acid sequence with the dual leucine zipper kinase (DLK) domain which signals via the MAPK cascade. The DKL is known for axon degeneration and neuronal apoptosis. Reports show that the DLK promotes axotomy-induced facial motoneuron death and excitotoxicity-induced hippocampal neuron death in neonates and adults respectively, acting as a known arena for neuronal insult. The LZK seems to be under-explored, although a study showed that LZK negatively regulates axon growth position downstream of Nogo (an inhibitor of myelin associated axon growth) [170–184]. Also, other reports demonstrate the positive regulation of axon growth in N2a cells and cerebellar granule neurons (CGNs). Although some studies indicate the suppression of DLK and LZK has a neuroprotective effect in the presence of the combinatorial treatment (Pten deletion + Zymosan + cAMP analog) their deletion suppresses axon growth [26, 185–188]. The DLK/LZK is involved in a kinase signaling cascade that elevates and triggers the activation of ATF2, cJUN, MEF2A, and Sox11. There may be cross-regulation between the DLK and LZK and their pathways may clearly outline the effect in response to injury [26, 185–188].

#### CNTF gene therapy modulation for regeneration

The role of the recombinant CNTF in inflammatory-induced regeneration happens to be viewed from a different perspective, however, its activities implicate it as a major mediator [158, 159]. Varying reports project the evidential effect and role of recombinant CNTF following the deletion of the SOCS3, a suppressor of the JAK-STAT signaling pathway [21, 165, 166, 189, 190]. The SOCS3 belongs to a family of eight-member proteins that impede the activation of STAT. Normally STAT is employed by growth factors, several cytokines and hormones for the transmission of information to the cell nucleus [191–193]. JAK upon binding to cytokine, activates its kinase under the (tyrosine kinase cytokine receptor interaction) to undergo auto-phosphorylation and after becomes bound to nearby heterodimer chain of cytokine receptor with different tyrosine interaction site in a cross phosphorylation. STAT via its SH2 domain then targets and binds these phosphorylated receptor sites of the JAK, this process becomes phosphorylated and further undergoes conformational changes to become bonded to other conformationally phosphorylated transformed STATS. The homodimer or heterodimer formed then becomes translated and channeled to the nucleus and targeted genes [193–195]. The SOCS3 regulates the activation of STAT3 via glycoprotein 130 (gp130) receptors in response to a cytokine and aside from that it is known to regulate response (cytokines, hormones, growth factors) which are gp130 independent (IL-12R granulocyte colony, leptin, granulocyte-colony stimulation factor (G-CSF), insulin, etc.) and does so to impede the activation of STAT3. The SOCS3 does not only employ signaling via the STAT protein but also through the MAPK or PI3 kinase pathways [196–199]. Although CNTF in gene therapy was reported to trigger robust regeneration, another factor that seems critical for the effectual function of the CNTF happens to be CCR2(a receptor that engages in recruitment of macrophages, C–C chemokine ligand type 5 (CCR5)). Wherefore, their deletion in the RGC causes the suppression of the CNTF gene therapy effect [28, 165, 200–206].

**Figure 3.**
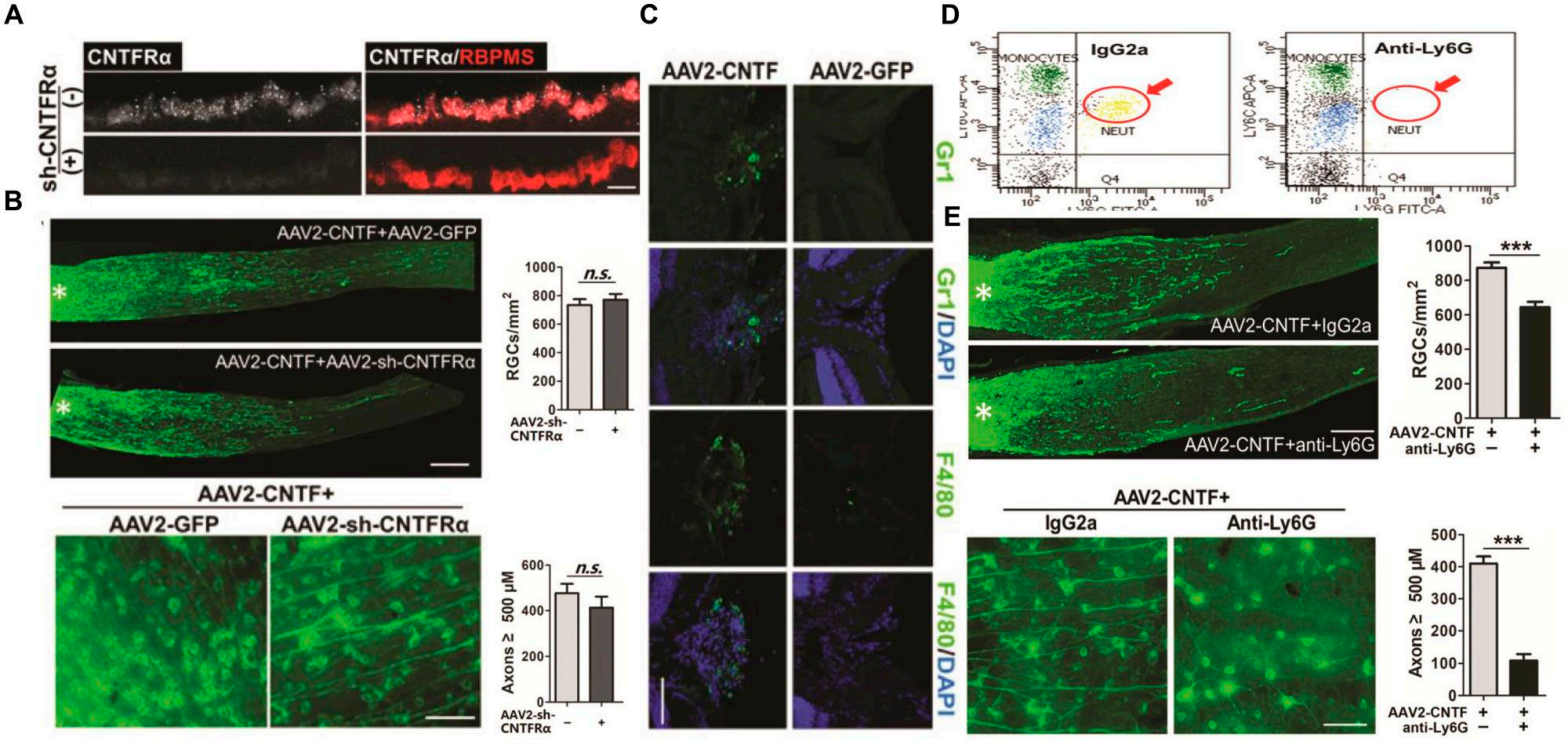
Activity of neutrophil in relation to CNTF gene therapy. (**A**) *In situ* hybridization detected low levels of CNTFRα mRNA (white) in the RGCs (stained with antibody to RBPMS (red) to delineate RGC cell bodies but not axon bundles). (**B**) Regenerating axons visualized by CTB immunostaining (green). The asterisk indicates the injury site, and the whole-mounted retinas immunostained with antibody TUJ1+ (green) to visualize βIII tubulin-positive RGCs. (**C**) Immune cells stained with F4/80 (green) (macrophages), Gr1 (red) and the nuclear marker DAPI (blue). macrophages (Gr1^low^F4/80^high^). (**D**) Immune cells isolated from blood; stained with fluorescently conjugated antibodies to CD11b, Ly6G and Ly6C. (**E**) Regenerating axons visualized by CTB immunostaining (green) and its quantification. Retinal whole mounts immunostained for βIII tubulin (antibody TUJ1) (green) 2 weeks after NC and quantitation of RGC survival. With approval, reprinted from Ref. [190]. Copyright 2021, PNAS.

The CNTF gene therapy in general thrives on the activation of the immune system together with CCL5 elevation. On this account, the selective deletion of the CCL5 receptor CCR5, or its antagonist hinders the effectual activity of the CNTF gene therapy. Also, it has been reported that recombinant CCL5 (rCCL5) enhances axon regeneration and RGC survival. The CNTF normally binds to CNTF receptor-α (CNTFRα), coordinating the formation of a tripartite receptor complex with gp130 and leukemia inhibition factors receptors-β (LIFRβ). Additionally, the CNTF is known to activate the phosphorylation of STAT3 in retinal neurons with RGCs inclusive, as the plasma membrane is anchored to the CNTFRα via a glycosylphosphatidylinositol linkage and through phospholipase C-mediated cleavage to become soluble [207–214]. Xie and coworkers reported that although CNTF was engaged in a tripartite receptor complex (gp130, CNTFRα, LIFRβ), the CNTF gene therapy was not affected following CNTFRα knockdown (KD), with negligible effect in the axon regeneration or RGC survival (Figure 3A and B). Hence the extrinsic expression of CNTFRα in the RGC under the CNTF gene therapy. Also, without alteration of the number of neutrophils, the CNTF gene therapy induced an increase in the number of macrophages thus enhancing of the infiltration neutrophils (GRI^+^) and macrophages (F4/80^+^) in the retina (Figure 3C). Neutrophils served as the immediate responders of the inflammatory cascade, providing the platform for the modification of the chemokine network and the influx of monocytes and broadly contributing to CNTF gene therapy [215–217]. Immune depletion of neutrophils strongly suppressed the activity of CNTF gene therapy and subsequently reduced axon regeneration (74%) and RGC survival (21%) (Figure 3D and E) [190, 205, 206, 218, 219]. A clear indication of the need for neutrophil activation for the effectual CNTF gene therapy.

### Signaling on the axon growth/regeneration

The signals for the induction of axon growth are also responsible for neuronal survival, making the differentiation of such signals a bit challenging. The over-expression of Bcl-2 can allow for the culture of RGCs in the absence of trophic signals, however, the removal of growth signal can impact the neuron, negatively. This indicates that the over-expressing of an anti-apoptotic protein still does not yield any axon or dendrite growth in the absence of a growth-inducing signal [220, 221].

#### Extrinsic signaling and intrinsic signaling on the axon growth/regeneration

Extracellular signals exist as peptide trophic factors referred to as neurotrophic factors which greatly influence axon outgrowth and are collectively called neurotrophins and comprise the brain-derived neurotrophic factors (BDNF), NGF, neurotrophin-3 (NT-3), neurotrophin-4 (NT-4) and neurotrophin-5 (NT-5). The different neuron responds to peculiar trophic factors although a number of them can be combined to trigger an efficient axon growth. A typical example is the greater extension of RGC axons in the presence of BDNF and CNTF [221, 222]. As extracellular signals are of great concern when it comes to the growth ability of the CNS neurons, so are the intrinsic growth factors. The possibility of the extracellular signals having an impact on the intrinsic signal is also a subject of concern. Although embryonic CNS is at a point able to regenerate their axons, over time this ability becomes retarded, with this largely associated with the maturation of glial cells, astrocytes, and oligodendrocytes [221, 223]. Also, an attempt to temper with any of the intrinsic abilities in some cases still leads to the loss of the regenerative ability. Hence, successful separation of the CNS neuron from the glial remains crucial for the changes that occur with the intrinsic axon growth ability. Further, due to intrinsic aging neurons, axon growth ability is affected yet elongates continually, this makes the subject of extrinsic signals impacting the intrinsic signals vague [220, 221, 224]. It is interesting to know that, there can also be a switch between the axon growth ability and that of the dendrite depicting the state of growth that will be evident even in the presence of an extracellular signal. In the presence of the aging neuron and the retarding of the growth of the axon, the extracellular signal effect is functional in the dendritic growth [221, 224, 225]. Further, studies show the intrinsic limitations could be overridden by the extrinsic factors among the CNS with other reports emphasizing the need for a Schwann cell that could clear byproducts of the myelin which is present in the PNS other than the CNS, thus a contributing factor to the inability of axonal regeneration in the CNS to occur [226–228].

#### Neurotrophic signals on the axon regeneration

Neurotrophic signal for axon growth brings to light some pathways, the peripheral neuron, for example, has the ras-raf-MAP kinase and PI3K/Akt signaling pathway at work. A blockade of both pathways completely blocks the axon’s elongation [220, 221]. Mostly under the blockade of Bax or the over-expression of Bcl-2, the pathways become operational and efficient, however, in the presence of NGF, a blockade of Bax together with either pathway still impedes the operational activity of the pathways preventing the axon elongation, thickening or even branching, as the different neurons may require different signaling requirement conditions. Aside from, the function of the pathways, the growth cone dynamics, the insertion of membrane and cytoplasmic building units is modulated by the neurotrophic signals [224, 229]. Also, neurotrophic signals are involved in the distribution of essential components of the neurons and induction of axon elongation via modulation of membrane supply and insertion. Further, they are involved in translation of axonal mRNAs [224, 230]. Hereinafter, emphasizing the fact that local protein synthesis could directly be regulated by such signals which can also induce the transport of mRNA, although the axonal distribution can easily be disrupted by the activity of an antisense oligonucleotide. Additionally, their role in the cell body has to do with triggering the transcription and translation needed for axon outgrowth [231, 232]. Interestingly other genes are equally upregulated emphasizing the link between cellular growth and axonal growth and the effect both conditions have on the axon growth [233, 234].

The responsiveness of a neuron to the neurotrophic factor is very key, it’s interesting to know that, the responsiveness is not the same for the CNS and PNS neurons. Normally neurotrophins are enough to trigger axon growth in the PNS. However, the contrary can be evident in the CNS even in the presence of CNTF and BNDF, without cAMP elevation. It’s worth noting that, the cAMP elevation alone does not also mean axon growth. But therefore, will require great electrical activity or even direct electrical stimulation. Consequently, these activities should be enough to modulate the responsiveness of the growth cones as well as the connectivity of the axons [235, 236]. This also emphasizes the difference in the ability of the CNS and PNS axon regeneration and the critical provision of the trophic peptides together with other signals such as the cAMP elevation or electrical activity for optimum growth [237, 238]. Furthermore, the use of neurotrophic support in RGC has been established, but there are concerns about the formation of new axons irrespective of neuronal survival. Hence the quest to probe the activation of growth promoting molecules such as mTOR and Janus kinase/signal transducers and activators of transcription (JAK/STAT) [239–241]. Another concern that has to do with the axon is the growth towards an aberrant target, thus the need for a condition to direct this growth. In this contest, cathode-directed growth *in vitro* has been phenomenal for this purpose. Next, is the electric field (ES) stimulation, an approach known to elude endogenous molecular directional cues and endogenous axon regeneration inhibitors *in vivo*. Another strategy is the fabrication of a scaffold that can equally be used in the axonal growth orientation and therefore will require a combinational approach factor signals for driving growth as well as a directional cue to properly direct the axons to their target [242–244].

### Mobile zinc (Zn^2+^) modulative role

Zinc which is essential for cellular function has gained widespread grounds, as it is required by a number of enzyme activities and transcriptional factors. Zinc mobile or chelatable zinc (Zn^2+^) is concentrated in some intracellular organelles and synaptic vesicles but with a concentration of ∼10^−10^ M within the cytoplasm. Zn^2+^ can either exist via metallothionein and intracellular organelles and can enter cells via voltage-gated Ca^2+^, specific Zn^2+^ transporters, Ca^2+^ permeable glutamate receptors, and transient receptor potential channels. Zn^2+^ within the synaptic vesicles of the brain enables synaptic transmission, coordinating neuromodulation and neuroprotection [245–250]. Zinc in hypoxic-ischemic injury results in neuronal death, interfering with ion fluxes, the mitochondrial, and other metabolic functions and contributing to the death of neuro and oligodendrocytes induced via oxidative stress. Also, zinc homeostasis abnormality may be crucial for understanding chronic neurogenerative diseases such as amyotrophic lateral sclerosis, Alzheimer’s, etc. [251–255].

#### Zn^2+^ chelation impact on the RGC survival

**Figure 4.**
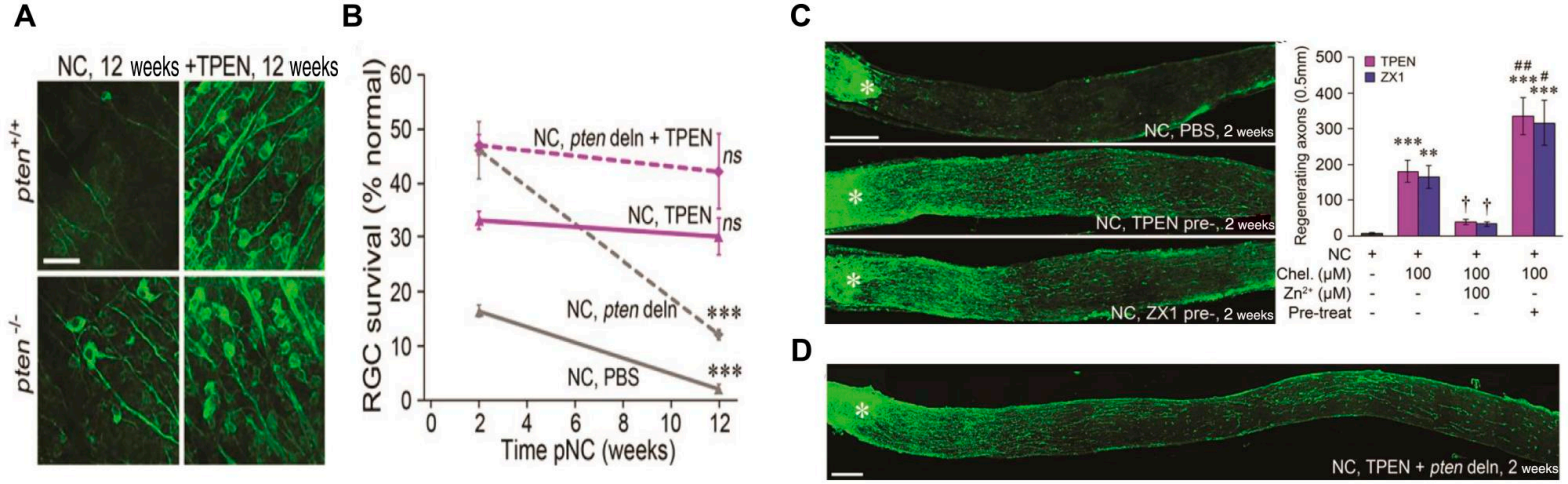
Zn^2+^ Chelation effect on RGC death. (**A**) Section of flat-mounted retinas immuno-stained (βIII-tubulin, 12 weeks. NC) for visualization of RGCs survival with/without TPEN treatment or *pten* deletion. (**B**) Quantitation of long-term RGC survival (**C**) Intraocular TPEN combined with *pten* deletion in RGCs for axon regeneration in mice with (**D**) Longitudinal sections of the mouse optic nerve immuno-stained (GAP-43 2 weeks. pNC) and its quantification. With approval, reprinted from Ref. [261]. Copyright 2017, PNAS.

A huge percentage (∼90%) of the zinc within the neurons end up as co-factors for enzymatic and protein structure related modulation via binding with metalloenzymes transcriptional factors and zinc containing proteins [256–258]. The rest (∼10%) exist as free for loosely bounding to proteins or as hydrated ions (free mobile zinc). Although emphasis has been placed on their importance, an excess amount can be detrimental. Chelation is a word of Greek origin ‘chela’ depicting a crustacean claw and a group of calipers-like units with a central metallic atom. The chelators bind to enhance excretion or decrease the metal ion with respect to their body concentration and have been employed for treating a number of degenerative disorders [259, 260]. Unsurprisingly, Zn^2+^ chelation increases the chances of RGC survival, injection with chelators such as the *N*,*N*,*N*′,*N*′-tetrakis(2-pyridylmethyl) ethylenediamine (TPEN) or ZX1 and the deletion of *Pten* (phosphatase and tensin homolog) suppressors of PI3K/Akt pathway enabled almost half of RGC survival emphasizing the need of the immediate blockade of the Zn^2+^ elevation following NC (Figure 4A and B). Similarly, on the axon regeneration, Zn^2+^ chelation influences the RGC effect on the regeneration as the mice group that was treated with intraocular TPEN or ZX1 early following NC demonstrated axons increase of about 25-fold, and also Zn^2+^ chelation with *Pten* deletion enabled some axon extension in just 2 weeks (Figure 4C) and even to the later end of the optic nerve (Figure 4D). Hence the Zn^2+^ chelation helps the RGC to impede and overcome a number of RGC-related death factors and therefore can be said to play a crucial role in RGC death. It should be noted that the Zn^2+^ amasses within the amacrine cells as retinal interneurons engage in the regulation of the injured RGC with considerable chances of RGC preservation for a prolonged period following Zn^2+^ chelation [261].

**Figure 5.**
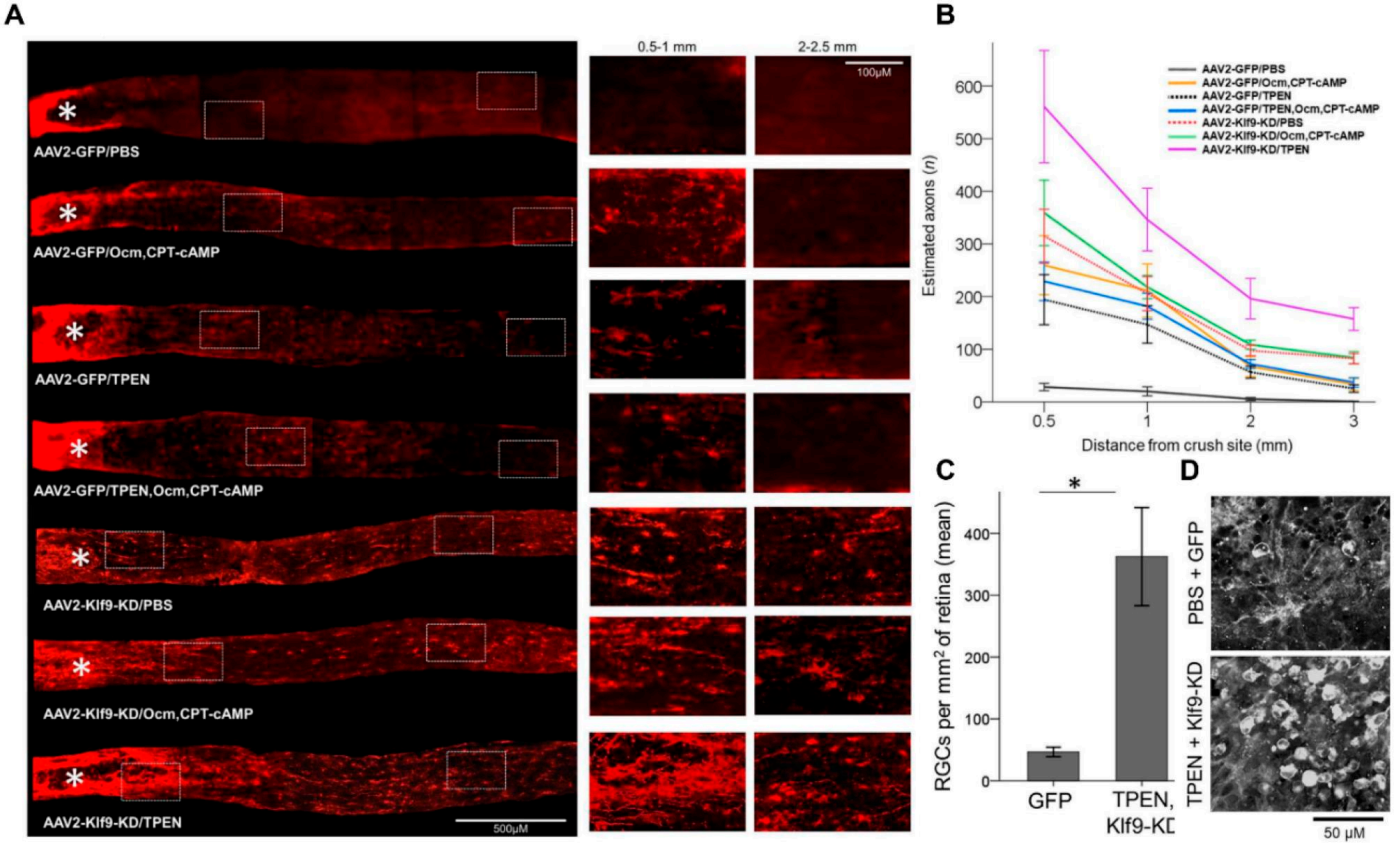
TPEN Effect on RGC survival. (**A**) Images of GAP-43-immunostained longitudinal sections through the optic nerve 2 weeks after ONC and magnified images of the optic nerve regions proximal and distal to the injury site. (**B**) Quantitation of axon regeneration 2 weeks after sole and combinatorial. (**C**) Nuclei marker (DAPI) stained horizontal section through the suprachiasmatic nucleus. Regions of the optic tract with CTB-labeled regenerated axons marked with dotted white lines. Absence of axons at, or growing towards the SCN. (**D**) Insets of the CTB-labeled axons in the optic tract regions as outlined in (C). With approval, reprinted from Ref [169]. Copyright 2018, Elsevier.

It is worth noting that the manipulation of several factors could potentially promote the regeneration and reinnervation of such site of target on the optic nerve, although concerns raised in association to such mechanism is the trigger of intraocular inflammation, knock-down or deleting of genes which may have some clinical implication [25, 153, 262–267]. Hence, Trakhtenberg and colleagues examined the combinatorial effect of TPEN and shRNA gene therapy mediated KD of the axon growth suppressing Kruppel-like transcription factor 9 (KLF9). Results from the combination of the KLF9 KD and TPEN under a 6-week study demonstrated substantially stronger regeneration as compared to when the KLF9 KD or TPEN were individually employed. (Figure 5A and B). Also, mice treated combinatorially depicted high levels of RGC survival (∼7) (Figure 5C and D). TPEN do not only chelate Zn^2+^ but can equally neutralize copper, cobalt, manganese, iron and calcium (divalent cation), the TPEN ensured gains in the survival of the RGC which was in tandem with a previous report of Li *et al.* Therefore, it could be said that the long-term RGC health and survival was due to the TPEN which at the same time enhanced the KLF9 KD effect for the long-distance axon regeneration [169].

#### The link of Zn^2+^ with the mitochondria

The pathogenesis relating to the RGC injury has been linked to some mitochondrial associated defects such as unfolded proteins response, decline of NAD^+,^ etc. Meanwhile, the RGC has been reported to be protected under treatments that were mitochondrial targeted, projecting the concern and possibility for the action of the Zn^2+^ to be mitochondrially linked since the Zn^2+^ is involved in a number of biochemical reactions of the mitochondrial. The Zn^2+^ could also be a point for the mediation of ROS production, movement and transport to other subcellular compartments or post-synaptic neurons, with excessive amounts causing other dysfunctionality processes of the mitochondrial and neuronal death [268–273]. The mitochondrial remains key in numerous internally systematic processes that are biologically related ranging from signaling, and metabolism to programmed cell processes such as (apoptosis) and ROS generation [274–277]. Also, within the inner membrane of the mitochondrial is the zinc metalloprotease which overlaps with the m-AAA protease 1 homolog (OMA1). The OMA1 upon activation becomes involved in the mediation of the proteolytic processing of the optic nerve atrophy protein (OPA1) which also engages in the crista maintenance, mitochondria fusion and generation of ATP [278–281]. Over-processing of the OPA1 is reported to promote the release of cytochrome C further leading to the activation of apoptosis. On the contrary, the silencing of OMA1 helps to attenuate a number of tissue damage. Also, the OMA1 in association with death-associated protein 3 (DAP3) could bind to cell death enhancer I (DELE1) to trigger integrated stress response following mitochondrial malfunction [282–288]. The DAP3 (mitoribosome subunit) engages in protein synthesis and mitochondrial homeostasis. Tang and coworkers elucidated the Zn^2+^ downstream mechanism (postsynaptic RGC somatic injury). It was reported that the activity of Zn^2+^ might be a downstream mitochondrial pathway that involves the activation of OMA1 triggering DELE1 cleavage in association with eukaryotic translation initiation factor 2α (elf-2α) kinase 2 protein kinase R (PKR) for the further trigger of the phosphorylation of elf-2α and the modification of the integrated stress response (ISR)-related gene. Studies indicate that this DELE1-ISR axis, with the activation of elf-2α, happens to be conserved in a number of diseases and neurodegenerative disorders with the OMA1–DELE1–ISR axis involved in the induction of RGC death following the accumulation of Zn^2+^ in an optic nerve injury. Hence the suppression of OMA1 could be done through Zn^2+^ elimination to blockade the activity of ISR in the RGCs for the RGC survival [289–294].

### Calcium ions (Ca^2+^) factor

High-level Ca^2+^ acts as a mechanical insult that compromises the cell membrane. It is well reported that intra-nerve scarring remains one key factor that impedes nerve regeneration, this scarred tissue retains a high amount of calcium ions which is a result of the sudden nerve injury. The Ca^2+^ ATPase becomes damaged and the Na^+^/Ca^2+^ assumes function in a reverse manner, facilitating the inflow of Ca^2+^ and the outflow of Na^+^, hence increasing the level of intra-axon Ca^2+^. These unbound intra-axon Ca^2+^ become involved in the activation of secondary Ca^2+^ dependent cascades which finally engages in the Schwann cell death [295–298]. Studies by Yan and coworkers depicted a negative impact on the survival and growth of Schwann cells by the level of Ca^2+^ correlated with those found in the nerve injury and concluded that Schwann cell survival and growth were negatively impacted by the elevated levels of Ca^2+^. Similarly, in their previous work, it was emphasized that the accumulation of Ca^2+^ had a strong correlation with the degree of nerve injury whereas functional recovery corresponded to that of the Ca^2+^ absorption. Although reports indicate the cellular process involving normal Ca^2+^ occurs at low levels, the overstimulation occurs as a result of the enormous Ca^2+^ amount. Mostly, the overstimulation of enzymes and other involving factors such as protein kinase, nitric oxide synthase, and phospholipase ends up activating some cell death-related signals or free radicals which are considered to be toxic, thus the damaging role of elevated Ca^2+^ during an optic nerve injury [299–304].

#### Ca^2+^ ions factor on the degeneration of the RGC

**Figure 6.**
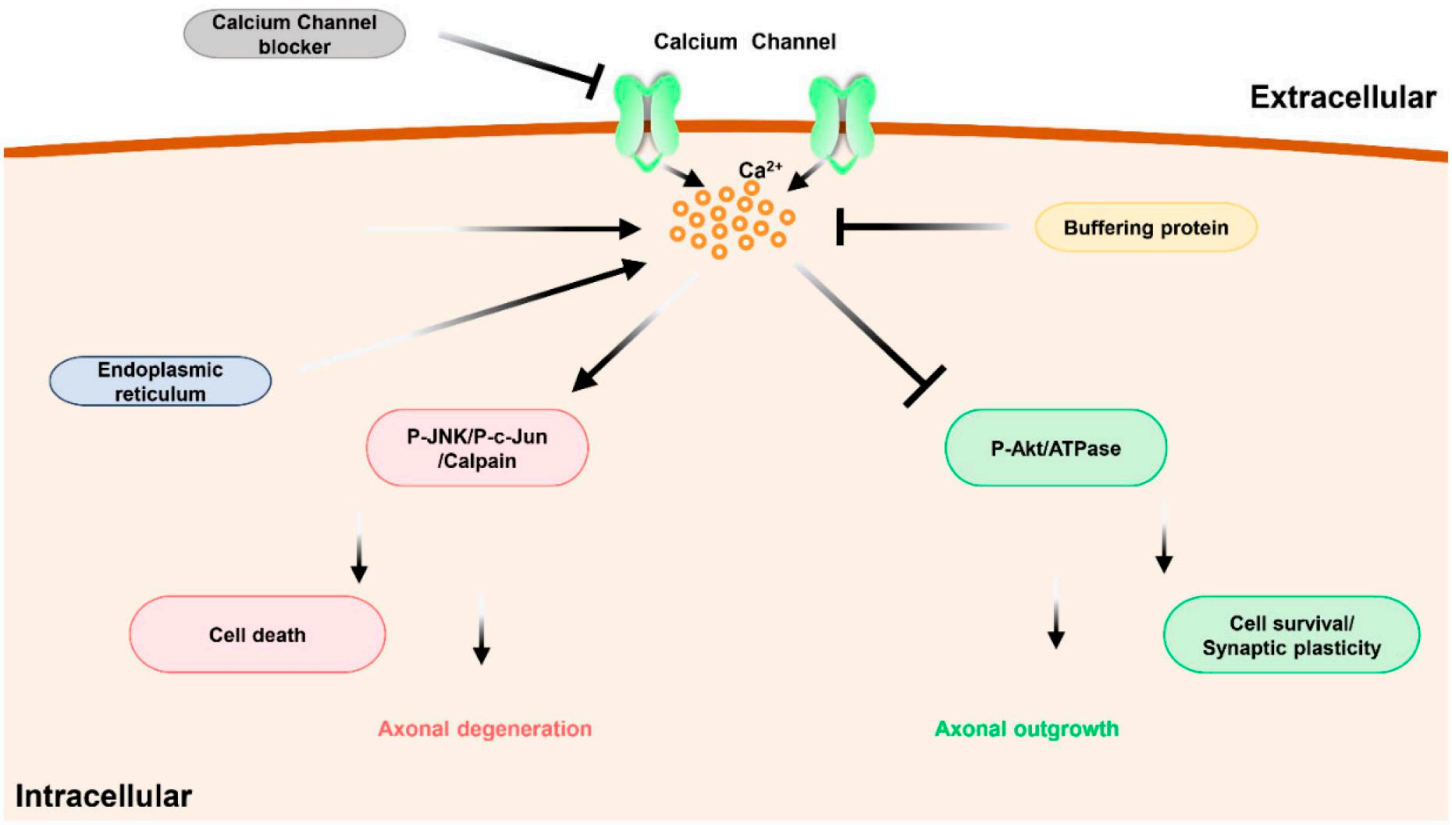
Schematic illustration of the inflow of the Ca^2+^ ions, cell death and axonal degeneration process.

The neural systems possess a homeostatic mechanism that regulates the level of intracellular and extracellular Ca^2+^. The diffusion of the Ca^2+^ occurs via a concentration gradient and is buffered by varying cytoplasmic potentials (parvalbumin, Calmodulin, Calbindin), by so doing the excess Ca^2+^ is expelled by the plasma membrane enzyme (Ca^2+^ ATPase) [305–308]. Hence the enormous localization of Ca^2+^ hastens the Schwann cells’ vesicular demyelization. Yan in 2009 reported that the degenerative cascade initiated by the elevated levels of Ca^2+^ is accompanied by nerve edema, demyelination, rupturing of axoplasm and vacuole formation which can collectively be observed under the Wallerian degeneration. Interestingly, because the degeneration of the axon is a somewhat self-destructing cellular process, sudden axonal disintegration is observed at the site of a focal traumatic lesion which extends on both sides. This degeneration axon term acute axonal degeneration (ADD) is characterized by the intra-axonal influx of Ca^2+^. Accumulating evidence suggests calpain proteases, downstream of calcium become activated and in the optic nerve for instance accompanied by the other calcium downstream mechanism such as autophagy. Consequently, the use of even autophagy inhibitors or calcium channel inhibitors has a great tendency to attenuate axonal degeneration [309–312]. Additionally, reports indicate that acute inhibition of calcium ion channel enhanced the axonal regeneration, just 28 days after ONC although this observation just occurs at a short distance with the inability to grow pass the injured site being attributed to the low intrinsic growth potential and other extracellular environmental factors. Employing the calcium channel inhibitors without addressing other inhibitory cues seeks not to entirely unleash the intrinsic axonal growth capabilities. Therefore, the requirement of an additional strategy for example the use of a calcium channel inhibitor has the possibility of easily reaching the superficial axons but not the inner optic nerve axons [313–316]. Also, JNK whose activation occurs under varying cellular stress, has a contributive role in the RGC, engaging in apoptosis and axonal degeneration. An elevation in its active form (p-JNK) can be observed even at the early phase of ONC, Ribas *et al.*, emphasized the reduction of p-JNK following the calcium channel inhibition. The various isoforms of JNK, JNK2 and JNK3 are associated with the RGC death and the JNK 1 and JNK 3 the axonal degeneration. Hereinafter, the possible link between the calcium channel inhibition and phosphorylation of these JNK isoforms. Also, another factor connected to the phosphorylation is the c-Jun. Interestingly, JNK activation within the RGC is known to be modulated by TNFα while the c-Jun engages in regulation of apoptosis of the RGC following ON lesion. Hence attenuation by the calcium channel inhibition holds the possibility of dealing with axonal degeneration and cell death (Figure 6) [316–321].

## The molecular achievement and the need for an alternative combined therapy

As mentioned earlier, the condition that succumbs the optic nerve to injury occurs through varying mechanisms which include traumatic optic neuropathy, optic neuritis, etc. Until now, it can be said that the unravelling of the entire mechanism and the progression of the above-mentioned conditions seems to be lacking. However, the molecular basis of the condition points to the fact that the RGC death ensues due to the inability of the RGC axons to reform and reach their original target [322, 323]. A large percentage of this death occurs via programmed cell death which indicates that during this period there are chances of overexpression of pro-apoptotic proteins. However, a surge in Bcl-2 expression tends to salvage the RGCs’ death [324–326]. The RGC death stemming from either a necrotic or apoptotic factor can be tantamount to perpetual damage of the optic nerve [327, 328]. Interestingly the extent of RGC death is largely determined by the distance and location of injury on the nerve tract in proximity to the eye. Also depending on the affected structure during the injury process, it can be either an axon or a cell body. Where it is the cell body, this further causes in access to targeted neurotrophic reinforcement, and under this circumstance the only alternative neurotrophic factor will have to be from an exogenous source example the BNDF, CNTF, etc. [329, 330].

Although the presence of this exogenous neurotrophic support does not guarantee a complete recovery but increases the chances of the RGC survival following injury. Usually, this exogenous neurotrophic factor is expected to be picked up by receptor sites located on the RGC, which unfortunately are mostly found on intact RGC. Hence causing the inability of the RGC to effectively respond to an exogenous neurotrophic factor [328, 331]. Over the years, some mechanisms that have been used to enhance this site receptor responsiveness or recruitment have either been the increase of trkB receptors, cAMP elevation, and subsequently effective electrical stimulation [332–334]. As discussed in the previous sections, several processes are involved during the injury of the optic nerve, and a number of factors are required in the attempt to help regenerate it. Since multiple therapies are required usually for the regeneration process. The next section will focus and provide details on the nanotechnological advancement which have yielded several biofunctional platforms that can be used to augment this molecular process or sometimes carry therapeutics for the salvage of the RGC and the regeneration of the optic nerve.

## Nanotechnological outlook for the optic nerve regeneration

The topic of regeneration of the nervous tissues seems to be perfectly appreciated under the PNS but that of the CNS and precisely the optic nerve seems yet to be fully unraveled. Nanomedicine employs physical and chemical features such as structure, size, molecular significance and intrinsic chemical properties for targeting and treatment of disease [335–340]. Their properties can also be modified to suit a particular target. Nanotechnological employment in such treatment has advanced over the years encompassing the engineering of cells, tissues, organs and the speeding up of research involving stem cells [341–344]. Under the stem cells, nanomedicine allows controllable regulation, differentiation, proliferation and migration, and even acts as nanocarriers for drug and gene deliveries [345–352].

In recent years, many biomaterial scaffolds have been used in eye treatments in general and are being embedded with cell-based therapeutics. These scaffolds allow for cell adhesion, release, migration and survival of cells. Based on the kind of biomaterial employed, they may be either natural or synthetic. However, there are concerns about the biodegradability and toxicity of these biomaterials [353, 354]. Mostly, these biomaterials are of organic source and biodegradable and do not necessarily have to be at the injured or damaged site for long to elicit any adverse effect or immune or body response [355, 356]. Also, that of the synthetic in most occasions per their design becomes hard to detect by the host immune system, hence allowing for the gradual release of any molecular materials like DNA, RNA, peptides, etc. These scaffolds being natural or synthetic can be made to be porous enough to create an optimal microenvironment while enhancing retinal axon stimulation [357–359]. Application of such combined or multiple technological material-based therapeutics is given in a subsequent section designed for enhancing differentiation and regrowth.

### Biomaterials

Further, the advances in nanotechnology have created the platform for the fabrication of new hybrid and synergistically functional biomaterials, among the biomaterials that have been of interest for soft tissue regeneration are polymers, two-dimensional materials, etc., aside from the earlier explored inorganic biomaterials such as the bioactive glass. However, their rejection by the body when used as implants sometime back, was due to the fact that synthetic materials do not respond to physiological or biochemical stimuli. Therefore, this challenge has been taken on by the nanotechnological industry to engineer materials capable of stimulating other signals and enhancing genes to initiate the process of repair or regeneration at the injured or damaged site [360–362].

#### Polymers

Polymers have a huge role to play when it comes to soft tissue repair or regeneration. Although those grouped as natural polymers such as alginate, chitosan, gelatin, polyester-based polymers such as polylactide (PLA), polycaprolactone (PCL), poly (lactic-co-glycolic acid) (PLGA), polyurethane (PU) can equally be used in the fabrication of materials or composite for both hard and soft tissues. Other polymer groups are the conducting polymers for mostly reinnervation purposes under electrical stimulation such as polythiophene (PTh), polypyrrole (PPY), etc. [363–365]. Aside from these functions, their usage in cell attachment, differentiation, migration or proliferation since their physical, chemical and structural features can easily be modified to suit that of soft tissues as mentioned earlier. Next, the combination of one or two forms of these polymers can also be used as a scaffold for tissue repairs. Interestingly, this has taken a widespread course ranging from several regeneration purposes among the skin, cardiovascular and nerve. Further, among the drug delivery vessels, hydrogel has gained a foothold due to its negligible toxicity, convenient delivery of novel drugs, and most of all compatibility with both small and large molecules making them an intelligent drug delivery system. Hydrogel themselves can exist in varying forms mimicking 3D tissue models with characteristics that present them perfectly as ECM. Considering their good mechanical stability, porous structure, good biocompatibility and their easy functionalization. They can take varying forms which include films, slabs, microparticles, nanoparticles and coatings [366–369]. All these features combine to demonstrate several biomedical purposes [370–374].

Hydrogel consists of a network of three dimensions, capable of evading solvating by absorbing a large molecule of water. They can exist as natural or synthetic and are considered soft materials. Natural hydrogels (gelatin, dextran, cellulose, chitosan and hyaluronic acid) and synthetic include polyvinyl alcohol, polyvinyl pyrrolidone, poly-N-isopropylacrylamide etc. [375–379]. Also, hydrogel can be categorized based on its crosslinking, process of production, derivatives, swelling features, provenience, ions or bio-recycling. Based on the crosslinking, the hydrogel can be considered to be physical or chemical. Those that have chemical crosslinking can be fabricated by either polymerization via irradiation, suspension polymerization, chemical reaction of functional group or enzymatic crosslinking. These crosslinks are developed between polymer chains by the crosslinking agents [380–385]. Also, the physical crosslinking requires either hydrophobic interaction, ionic interaction, protein interaction or hydrogen bonding crystallization. However, chemical hydrogels based on their crosslinking are irreversible owing to their configuration. Based on their structure, they can be crystalline, semi-crystalline, amorphous or hydrocolloid aggregates. Based on ions, they can either be cationic, anionic or neutral. Furthermore, based on the combination of the electrostatic interaction with the physical or chemical characteristics, they can be considered as double network hydrogel. Also, based on their responsiveness they can be classified as stimuli-responsive hydrogels, transforming electrical, chemical, magnetic, mechanical or optical stimuli to other forms of signal, which is evident in mechanical strength networking, growth action and permeability [386, 387]. These forms of hydrogels are simply referred to as smart hydrogels. Among these hydrogels are those that are temperature sensitive and a typical example is the *N*-isopropyl acrylamide and methylcellulose. Under this condition, they demonstrate hydrophilic characteristics as a result of the hydrogen bonding between the H_2_O and the pendants groups even at a lower critical temperature of a solution (LCST) and breaking the bond at a higher temperature to become hydrophobic. Similarly, others are able to ionize in response to pH.

Additionally, the porous nature or structure of a hydrogel is dependent on the pore size or porosity measurement. This also correlates to the magnitude of the distance between polymer chains employed for the bioactive agent diffusion at a point. The extent of swelling and the release of bioactive molecules is dependent on the porosity of the network structure which is also dependent on the density of cross-linking. These affect the rate of loading and the release of a bioactive agent. Also depending on the pore size of the hydrogel, they can be categorized as microporous (100–1000 µm), macroporous (0.1–1 µm) or non-porous (10–100 µm). Biological fluid or water penetration between this crosslinked network is observed as the swelling in the hydrogel, giving it the soft tissue-like mimicry for the integration with tissue and at the same time minimizing the tendency of irritation due to their soft and rubbery nature [388, 389]. Importantly, the influence of the mechanical and absorption properties effectively depends on the cross-linking. Although crosslinking does not effectively occur when the crosslinking agent is low, however, an increasing amount of the crosslinking agent contributes largely to the development of other network structures stemming from the large numbers of aggregated polymer chains. Under such situation, there is a decline in the swelling ratio as the crosslinking agent modulates the crosslinking density. This can occur either *in vitro* (under the hydrogel preparation process) or *in vivo* following their application in their target location [390–395].

The movement of cellular and other products within the hydrogel is also dependent on the state of the water incorporated and retained within the hydrogel interaction between the water and the groups with maximum hydrophilicity (primary attached water). This interaction initiates the swelling process, exposing the hydrophilic network which leads to the formation of bonds that are hydrophobic (secondary attached water). The hydration of both polar and non-polar regions of the hydrogel builds an osmotic pressure that allows further absorption of water. These primary attached water and secondary attached water are considered as the total bond water [393, 394, 396–399]. It is worth noting that most of the time the gel tortuosity (pore distribution, pore size, and interaction existing between pores) makes it challenging to measure the gel network. Also, most times, the drug matrix polymer relation, diameter of drug molecules and small pore size do not permit the permeation of the drug. Hence in overcoming this challenge in the designing of a hydrogel for the effective release of a drug the polymer composition and the thickness of the cross-linking should effectively match the size and composition of the drug expected to be delivered [400–402]. In the absence of water or a biological fluid, these hydrogels exist as xerogels or aerogels without any structure deformity except for the absence of water. Also, although their network backbone contributes largely to their physiological nature and morphological state, they happened to be altered under some conditions such as (temperature, electric field, pH and other reactions such as hydrolysis) [403, 404].

#### 2D materials

2D materials are materials whose electron movement on a nanoscale is considered to be in two-dimension with their unique features dictating their biological, chemical and physical properties. These 2D materials include transition metal dichalcogenides (TMDS), metal-organic framework (MOFs) and graphene, together with other 2D nanomaterials that are self-assembled. The TMDS’ unique electric ability has huge importance in neural repair. They promote neural stem cell (NSC) differentiations in the direction of neurons and neuroglial cells and act as a platform for the cultivation of neural cells [405–408]. Among them, the layered double hydroxides (LDHs) (anionic clays), have modifications of the chemical make-up which allows their easy screening towards specific research needs, and are normally synthesized under either induced hydrolysis, ion exchange, or co-precipitation. This synthesis approach largely determines the chemical composition, shape, size and state of aggregation [409–412]. On the topic of regeneration, LDH can be used for drug delivery and are able to release drugs gradually owing to the intercalation of the drug within them, and also have immunoregulatory functions. Also, on the basis of their biocompatibility, a known LDH (Talcid) was approved for the treatment of stomach ailment induce gastric acid accumulation thus, the clear indication of the possible control of both the physical and chemical properties for the successful regulation of the toxicity that a nanomaterial may pose. Under some physiological conditions or acidic environments, they can decompose or dissolve, decreasing the chances of risk of their accumulation. Also, toxicity associated with the LDH is reported to be based on the form of the cell that is being used. A typical example is its negligible toxicity effect on the activity of RBC cells, human umbilical vein endothelial cells and vascular smooth muscle cells [413–417].

Also, another 2D material extensively employed under regeneration is graphene, a carbon nanomaterial with a hexagonal honeycomb structure consisting of carbon (monolayer) with the covalent σ-bond existing between individual atoms and three other adjacent atoms and comprising of a number of derivatives (graphene quantum dots, reduced graphene oxide (rGO), graphene nanosheet, together with other graphene’s, few layer graphene and monolayer graphene) [418–421]. The complexity of the material largely depends on the form of synthesis. The wide usage of this material in neural repair and regeneration will have to do with their excellent electrical property making them suitable for electrical stimulation which is required for enabling the regeneration of excitatory neurons. Aside from that they allow functional integration and survival of transplanted cells through the provision of structural support. Also, another role played by graphene-based nanomaterials is their ability to be employed as nanocarriers, presenting drugs for neural regeneration. Although mentioned as efficient for drug delivery and electrical signals, one limitation associated with the material is its inability to exert bioactivity on neurogenesis due to its hydrophobicity, hence the need not to be solely employed for neural regeneration [422–427]. Further, extensively used with graphene-based materials are chitosan, collagen, PLGA, etc. They can serve as an interface for the improvement of neuronal regeneration [428–432].

Another 2D material is black phosphorus (BP) which consists of a number of layers and crystal structures that are orthorhombic permitting the covalent bond between the phosphorus atom and other three adjacent atoms, as observed in the graphene, with their structure appearing as a puckered honeycomb. The BP has a high anisotropy although it can be changed in accordance with the strain application, with its stacked layer defining a number of its features. Next, their conductivity paves the way for their use in tissue engineering. BPs have good biocompatibility and biodegradability and can also endow a hydrogel with conductivity and mechanical strength. The high specificity of their surface area ensures their efficient loading of drugs. Further, the BP coverage for a wide range of light (UV to strong light) and hence crucially in postsynaptic action (excitatory and inhibitory), makes them ideal and have potential for neural systems owing to their synapse-like features [433–437]. On the topic of cytotoxicity and biocompatibility, due to their inherent degradability, they can degrade with negligible toxicity. Since the final product (phosphate and phosphonate) of the BP are non-toxic. Additionally, the amount of material, time of exposure, rate of degradation and the type of cell can also determine if there will be a possible induction of ROS, DNA damage, apoptosis or autophagy when employed, tissue damage and inflammatory reaction can occur as a result of acute exposure [438–443]. Also, MXene like other 2D materials has been used for neural photothermal excitation due to their excellent photothermal conversion efficiency. However, their application under this condition needs the uttermost attention and care to prevent pathological abnormality or irreversible damage arising from the illumination with a high-energy pulse. Characterizing the local photothermal response with micropipette-based thermometry, Wang Yingqiao examined the photothermal excitation of neurons with a single flake and thin film Ti_3_C_2_T_X_ and demonstrated the tight soma adhesion and extreme neurite growth following culturing on Ti_3_C_2_T_X_ film [444, 445].

In general, the use of the nanoparticle is purposely for enhancing mechanical stability and allowing for the perfect and controlled release of drugs. On most occasions, the graphene and other 2D nanoparticles act efficiently when also combined in this manner. A typical example is the incorporation of CNTS (carbon nanotubes) or carbon-based nanomaterials since they are known for their ability to enhance hydrophilicity and excellent electrical conductivity. Also, other forms of nanomaterials that have been incorporated in the hydrogel are the combination of both metals and metal oxides, forming hybrid nanomaterials that can display electrical and magnetic abilities for their use in the biomedical field especially tissue regeneration. Aside from, the regenerative function they can also be employed for biosensing, bioactivation, delivery and bioactivation [377, 446–449]. Further, another group of nanoparticles that have been extensively used in hydrogel incorporation is the inorganic nanoparticle (CaP, bioactive glasses, silica, ceramics, etc.), for modifying the mechanical properties and possible re-enforcement [450–453]. Also, CaP has been employed for periodontal tissue regeneration, calcium silicate (CS) has also been effectively used for tissue repair and further supporting collagen type 1 deposition, allowing for wound healing, proliferation, migration and differentiation of cells, neovascularization, articular cartilage, adipose tissue growth and angiogenesis [11, 454, 455]. The bioactive glass (BG) has also been utilized for drug delivery and is able to release its constituent metal ions upon contact with fluids for anti-inflammatory, antibacterial activity, cell proliferation and angiogenesis, regeneration of a facial nerve in a sheep and even in reinnervation [456–458]. Although some modifications are needed to ensure perfect chemical composition, they have largely demonstrated significant effects compared to other forms of biomaterials employed for conduits fabrication [459, 460]. Fast forward, to the discovery and fabrication of nano scaffolds that can mimic 3D extracellular matrix (ECM), equipped with mechanical properties, conductivity, stimuli responsiveness or even antibacterial properties. These nanotechnologically developed scaffolds directed towards regenerating the nerve can exist as nerve wrap (NW) or nerve guide conduit (NGC). The nerve wrap consists of a sheet of material (rectangular cut shape) that can be folded, rolled into a tube or rolled around the approximated nerve stump and secured in its position by either glue or suturing, similarly, the nerve conduit consists of an already tubularly made material in which the proximal and distal nerve stumps are directed and are held in place by a glue or suturing [461–463].

### Nerve guide conduit

**Figure 7.**
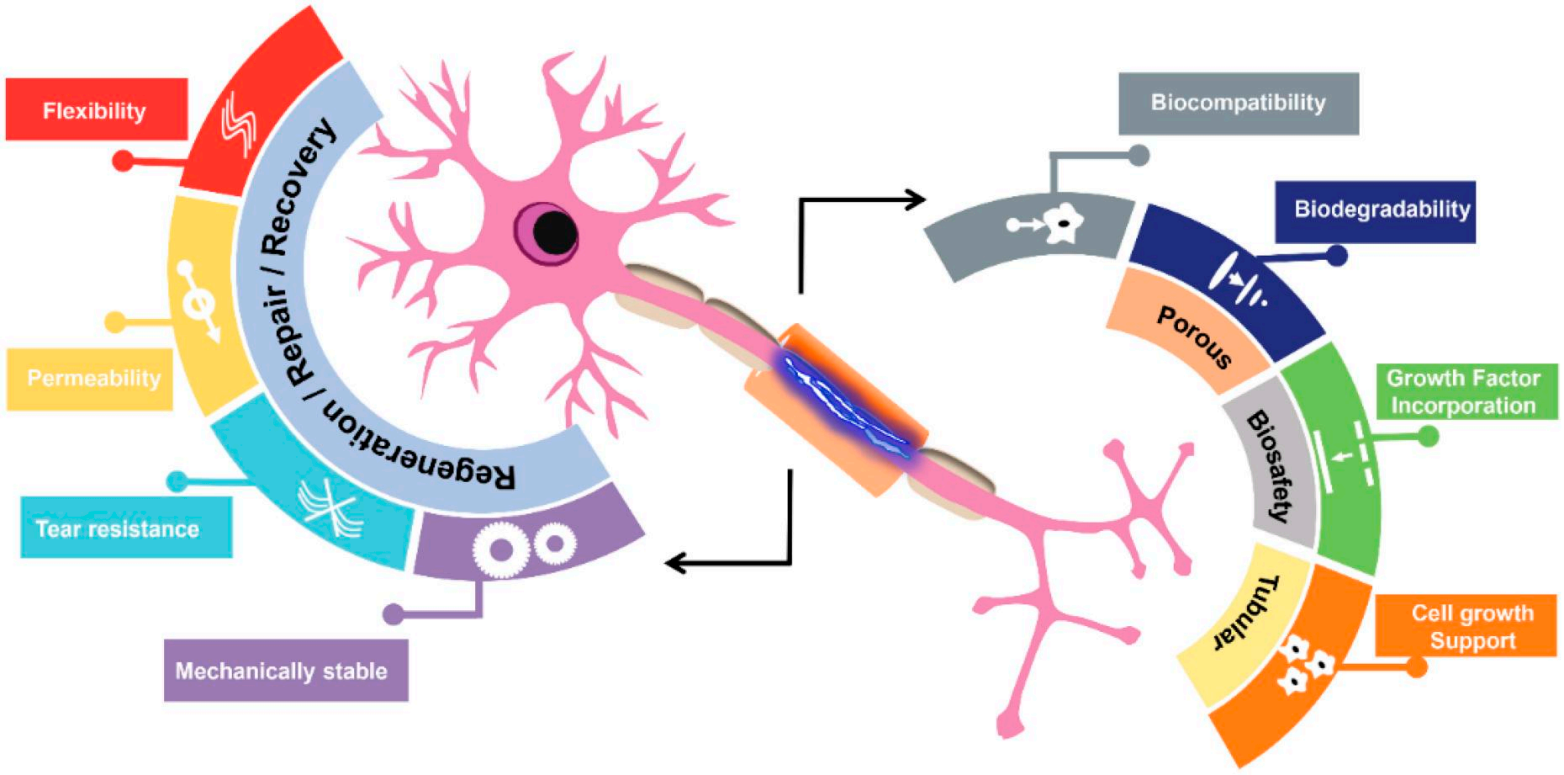
Diagrammatic representation of features of a suitable NGC.

The concept of autograft which has been one of the widely known approaches for regeneration has continuously been explored although not without setbacks. In clinics, the emergence of commercial NGCs such as Neurotube, Neurolac, Neutrogena, etc. has still not yielded any substantial development in the field of optic nerve regeneration. These challenges have been associated with the mechanical cell-related properties (proliferation and differentiation) and, the absence of complete directional nerve regeneration support making them very difficult to employ for the optic nerve regeneration. Further, other challenges include the inability to regenerate longer nerve gaps as the majority are limited to short-distance nerve gap regeneration [464–470]. Another limitation is the inability to act as an electroactive substrate thus not being able to be employed for or under electrical stimulation for neuro-generative purposes. The topic of electrical conductivity brings to light the connection existing between the conductive scaffold and the electrical stimulation, period, and dosage [471–474]. However, with time these challenges may be overcome as evident in a number of recently fabricated NGCs. A typical nerve wrap or nerve conduit (i) should be nontoxic and biocompatible within its cellular environment inducing no inflammation or infection. (ii) should be permeable, porous to a certain extent, and biodegradable, allowing for the optimum nutrient and oxygen exchange, large pore NGC (∼20–25 µm) permit axon regeneration compared to smaller pore size, taking into consideration the easy diffusion of growth factors and possible prevention of inflammatory cells infiltration. (iii) should be flexible yet mechanically stable for the resistance of tensile bending stress and tear during suturing [461–463]. (iv) should be tubular or easily rolled into a tubular state with the internal structure made directionally spacious enough to allow growth without compression and misdirection. (v) Should be stable enough to be loaded with different biological cues (growth factors, fibrin, other extracellular elements such as laminin, fibronectin, collagen, etc.) or can be electrically conductive since this facilitates neuronal conductive movement and reinnervation, hence a device with loaded biological cues or a device's design to be electrically conductive (Figure 7) [475–484].

#### NGC with/without biological cues

**Figure 8.**
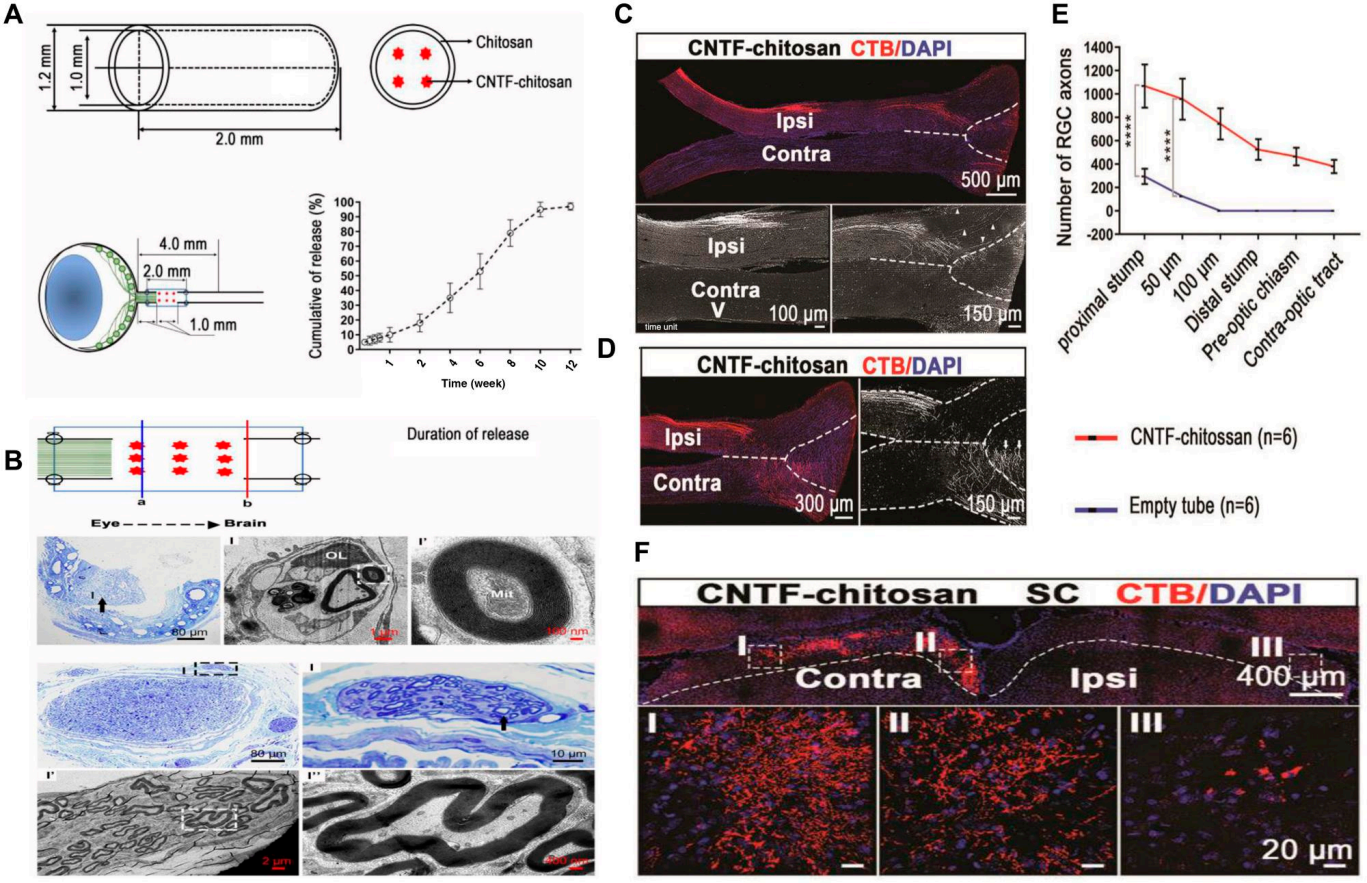
NGC With some biological cues. (**A**) Diagrammatic representation of the optic nerve surgery and the release kinetics of CNTF. (**B**) Images of toluidine blue staining and the transmission electron microscopy of cross-section at the Middle part of the lesion site and the distal optic nerve stump. (**C**) CTB-labeled RGC axons from the CNTF-chitosan group. (**D**) The axons in the ventral hypothalamus. (**E**) The number of the regenerated axons as a function of distance from the proximal stump of the optic nerve. (**F**) CTB-labeled axons in the SC. High-magnification images of the marked regions. With approval, reprinted from Ref [501]. Copyright 2023, Springer Nature.

Interestingly some nerve conduit functionality and use have been reduced due to the limitation of not being biodegradable or being biologically inert [485–487]. Normally optimal regeneration of the nerve is achieved when the growing axon traverses the scarred repaired section to the distal segment since fibrosis and glial scarring act as blockades to axonal regeneration through growth rate impediment and the regrowth of axons [57, 488–491]. Wherefore, employing a neuro-protective approach could suppress or help to overcome this fibrosis formation setback at the repair sites. This emphasizes the criteria for a good nerve wrap which will have to be biologically inert, biodegradable, and should provide conditions for the prevention or blockade of intraneural scarring at the repair area [492–497]. Liu and coworkers employed chitosan in the fabrication of the tube (conduit) loaded with CNTF and chitosan (Figure 8A). They demonstrated that the CNTF-chitosan was able to ensure the reconstruction and functional recovery of the visual system of an adult rat. The NGC could release CNTF at a physiological temperature for weeks providing a conducive environment for the long-distance axon regeneration of the RGC with the RGC axon passing the lesion site and projecting into the distal optic nerve stump. More importantly, the myelination preceding the regeneration of the RGC axon (Figure 8B). The challenging factor under the regeneration is the passing of RGC axons through the optic chiasm with some regenerated axons exhibiting axonal turn back and most reaching the contralateral optic tract while few got to the ipsilateral tract via the optic chiasm (Figure 8C and D) and finally, the resolve to restore the visual function. CNTF-Chitosan was shown to cause the projection of regenerated axons into the bilateral optic tract with its termination in the visual nuclei seated in the brain (Figure 8F). With the demonstration of nascent axons from the spared axons [498–501].

**Figure 9.**
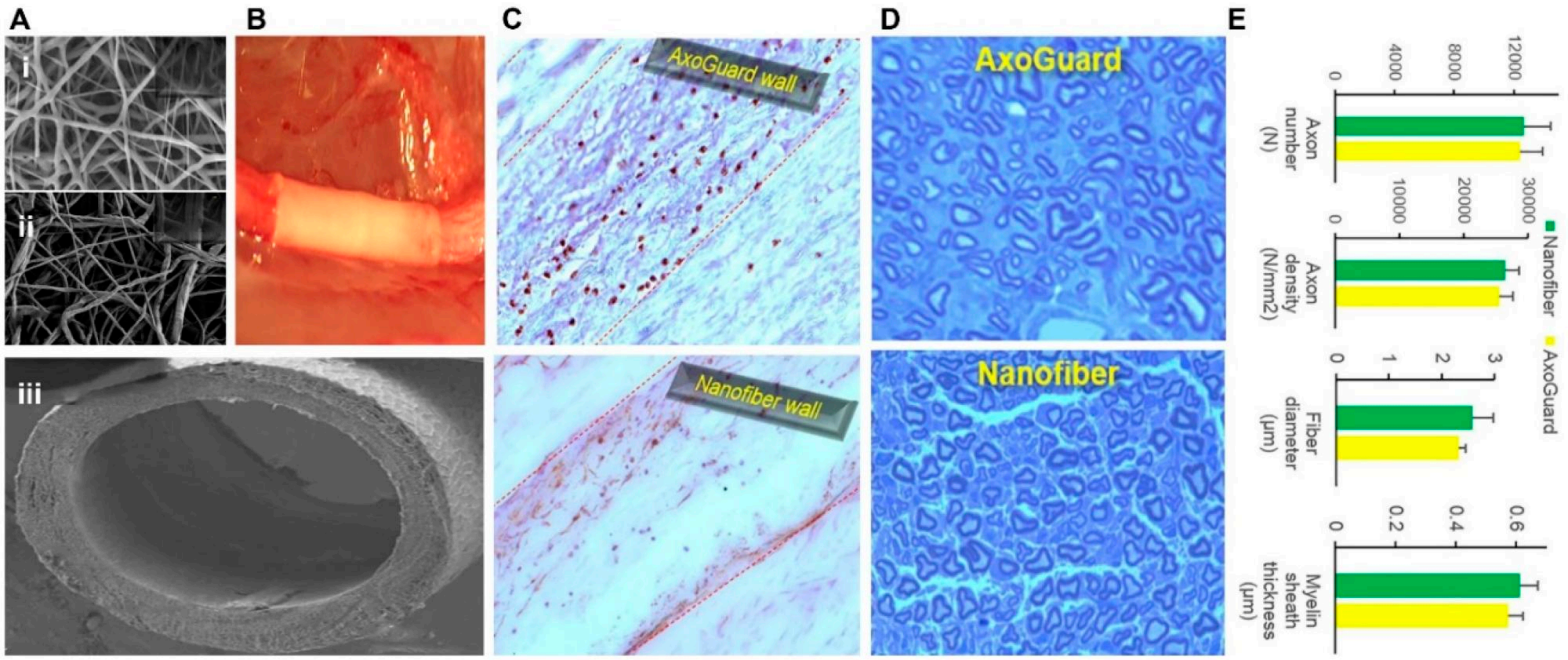
Function of NGC without biological cues. (**A**) Pre- and post-heat treatment SEM images of the poly(e-caprolactone) nanofiber wrap. The scaffold has a three-dimensional, interconnected pore structure and consists of randomly oriented fibers. (**B**) Gross examination of the repair site at 5 weeks post-repair. (**C**) The denser inflammatory response elicited by the AxoGuard is evidenced by the enormous number of macrophages that invaded its wall as compared to the nanofiber wrap. (**D** and **E**) Comparing the number and density of axons in the AxoGuard group as compared to the nanofiber group. With approval, reprinted from Ref [513]. Copyright 2019, Elsevier.

Over the period, another challenge faced with the fabrication of nerve wrap is the contracting effect which prevents nerve gliding and vascularization with a high inflammatory response [502–504]. Also, due to the fact that exogenous stem cells are faced with some limitations such as rejection response, limited cell survival and inadequate differentiation. Consequently, in overcoming both setbacks endogenous cells have to be relied on for regeneration and this requires a bioactive scaffold that will help in the recruiting of endogenous cells to the injured site and at the same time present a suitable environment for the differentiation of cells in suitable neurogenic lineage [505–512]. Sarhane and coworkers fabricated a nerve wrap of nanofiber via electrospun to give a scaffold with high porosity, high tortuosity interconnected pores and having the inner and outer wall presenting biomimetic platform. The soft and pliable nature of the material allowed for perfect nerve gliding without difficulty, and demonstrated negligible constrictive effect following animal sacrifice (Figure 9A and B) [513]. In comparison with AxoGuard nerve protective wrap whose morphology is somewhat smooth and planar and consists of several layers, the fabricated nerve wrap had a macroporous structure. Examination of the degree of fibrosis together with the axonal regeneration showed similarity in the mean number of intraneural macrophages and the deposition of intraneural collagen among the two wraps with no significant difference existing among them (Figure 9C). Also, the macrophage invasion of the AxoGuard wall was quite enormous compared to nanofiber wrap which was suggested to be the level of inflammatory response elicited at the repair site. Similarly, no significant difference existed between the histomorphometry parameters of the two-nerve wrap declaring statistical similarity between the two-nerve wrap for the number and density of axons. (Figure 9D and 9E) [513]. In a typical NGC application, it can be said that either a biological cue is employed or some form of stimulation is induced in the NGC to ensure effective functioning for regeneration. Examination of most NGC ranging from those applied in the PNI and recently CNI shows a vast majority have either a stimulation (electrical, photoelectric, ultrasound or sonodynamic) that is (in the absence of growth or other factors) or a combine’s suitable polymers with part used in the fabrication whole conduit and other part used as fillers or lining the inner section of the conduit or wrap that is (when stimulation is absent) (Table 1).

**Table 1.** NGC Modulation of nerve regeneration with growth factors or other stimulating effect

Material	Nerve type	Usage	Stimulation	Growth/Other factors	Ref.
PGA-chitosan/L1-Fc	Optic nerve	Axonal regeneration/remyelination	Absent	Present	[514]
CNTF-chitosan	Optic nerve	Regeneration and functional recovery	Absent	Present	[501]
PCL/collagen/rGO/PCL	Sciatic nerve	Neovascularization/M2 transition	Electrical	Absent	[515]
A/G-PCL	Sciatic nerve	Nerve defect repair	Absent	Present	[516]
r(GO/GelMA)	Sciatic nerve	Potentiate regrowth/myelination	Electrical	Absent	[517]
CSF-GNDF	Sciatic nerve	Neuron protection/reinnervation	Absent	Present	[518]
PLLA/mMWCNT	Sciatic nerve	Increase the number of axons	Absent	Present	[519]
PLGA	Sciatic nerve	Reinnervation	Absent	Present	[520]
PA	Sciatic nerve	Re-bridged neurofilament	Absent	Present	[521]
CNT/sericin	Sciatic nerve	Target muscle reinnervation	Electrical	Absent	[522]
Silk nanofiber	Sciatic nerve	Regeneration	Absent	Present	[523]
Chitosan	Sciatic nerve	Distal target reinnervation	Absent	Present	[524]
Ns/chitosan	Sciatic nerve	Block apoptosis/cellular migration	Absent	Present	[525]
Collagen coated (PGA)	Sciatic nerve	Prevention of neuroma	Absent	Present	[526]
Silk/sericin/silicone	Sciatic nerve	Improve nerve function	Absent	Present	[527]
Sericin	Sciatic nerve	Schwann cell proliferation	Absent	Present	[528]
PLCL	Sciatic nerve	Vascularization/regeneration	Absent	Present	[529]

#### Electrical stimulation of NGC

The electrical stimulation of a scaffold provides a platform for the mimicking of the electrically conducive micro-environment of the nerve. Due to the electrically active state of nerve cells, electrical conductivity remains crucial in the regeneration, cell movement, proliferation, and differentiation [475, 476, 530]. Studies indicate that electrical stimulation (ES) engages the release of NGFs, sprouting axons and sensory axons reinnervation [531–533]. Further, electrical stimulation allows the upregulation of the protein involved in regeneration and axonal growth. Initially, this was done via the implantation of an electrode at the site of the defect followed by the induction of an electrical stimulation from an external source of power supply [534–536]. Some conditions for the use of ES platforms are the sustained and controlled power supply, being in the state to adapt to cell culture conditions, pulse, and digital simulators, measuring systems, electrodes (stainless steel, Au, etc.) for the external power, and potentiostat. Usually, chemical neurotransmission is induced by a potentiostat, and the electrodes via an electrochemical process respond to this neurotransmission. Fast forward this stimulation reaches the nerve cells via electron transfer [537–550]. The condition that happens to hinder in-depth studies at this stage is the know-how of the link between the NGC, conductive materials, and neural cells, another challenge is the post-operative associated means of dealing with microelectrode, the injury, trauma or inflammation that may develop after. Although at the time of implementation, this seems quite effective, over the years, the requirement of additional surgical means for the disconnection and removal of the electrode after the injured nerve is healed can cause other complications such as secondary injury, and may be strenuous. Also, another limitation faced with the approach is the uncontrolled mechanical and conductive properties coupled with poor processability [551–553]. Howbeit, in recent years, the emergence of electro-active scaffolds that employ conductive materials or conductive polymers mainly gained attention because of the possibility of overcoming earlier limitations. Electroactive scaffold remains valuable and operational towards electroactive cells (neurons and muscle cells). However, the optimal function of the ES is dependent on the state and extent of application of the electric conductivity [554–559].

#### Photoelectric stimulation of NGC

**Figure 10.**
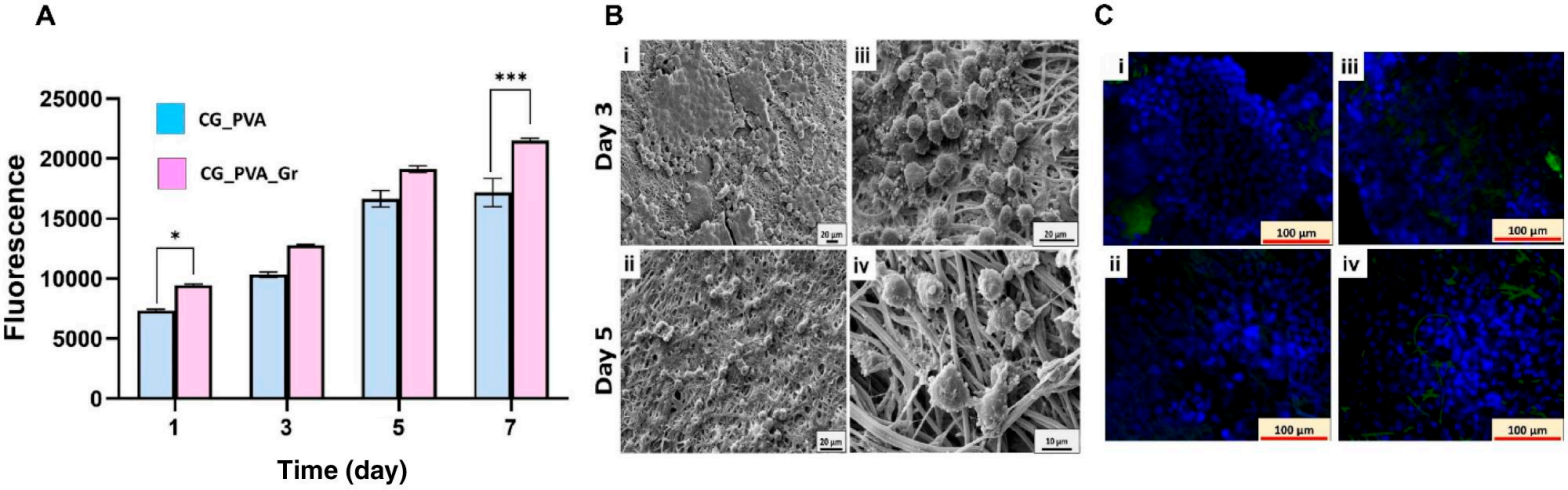
Neural cell communication from photoelectric cues. (**A**) Biocompatibility evaluation of CG_PVA and CG_PVA_Gr electrospun sheets on neuro 2a cells. (**B**) SEM images of the morphology of neuro 2a cells on (i, ii) CG_PVA, (iii, iv) CG_PVA_Gr nanofibrous sheets on day 3 and day 5 of culture. (**C**) DAPI staining of neuro 2a cells on (i, ii) CG_PVA, (iii, iv) CG_PVA_Gr nanofibrous sheets. With approval, reprinted from Ref [563]. Copyright 2023, Elsevier.

The discovery of photoelectric materials inspired the generation of photocurrent as an excitation from an external light source, doing away with the strenuous approach of carefully wiring, insertion, and removing electrodes [560, 561]. These photoelectric materials have also gained widespread attention owing to their high absorption coefficient, narrow band gap and excellent photon conversion efficiency with good biocompatibility. Srivastava and coworkers employed the nerve wrap model in combination with electrical stimulation for a median nerve injury in rats. The work of the electroband was to target three key areas that serve as the foundation for the regeneration of the nerve, neuroprotection, neuroregeneration and neuroplasticity, providing the platform as an extracellular matrix, the reconciliation of the injured environment while preventing anoikis (a form of apoptosis). In their work, rGO functionalization in combination with intraoperative electrical stimulation aided the injured nerve in attaining its denervated target with the growth of nerve fibers and neuroplasticity. Also, there was 80% regain of muscular strength within a few weeks. Interestingly, the biocompatibility of the electrospun electro band with Neuro 2a cell culture portrayed under resazurin assay, an enhancement of a significant cellular adhesion and proliferation of the Neuro 2a cells, unraveling the efficacy and biocompatibility of the rGO, hence the needless utilization of peptides and proteins in the functionalization (Figure 10A). Also, SEM images of cells on the days 3 and 5 showed the morphology and proliferation of cells among the rGO functionalized electrospun sheet and further demonstrated that the Neuro 2a cells portrayed a significantly dispersed form of growth, depicting the importance of the electrical cues for the neural cell’s communication (Figure 10B and C). More importantly, emphasizing the enhancement of the scaffold bioactivity following functionalization [562, 563].

**Figure 11.**
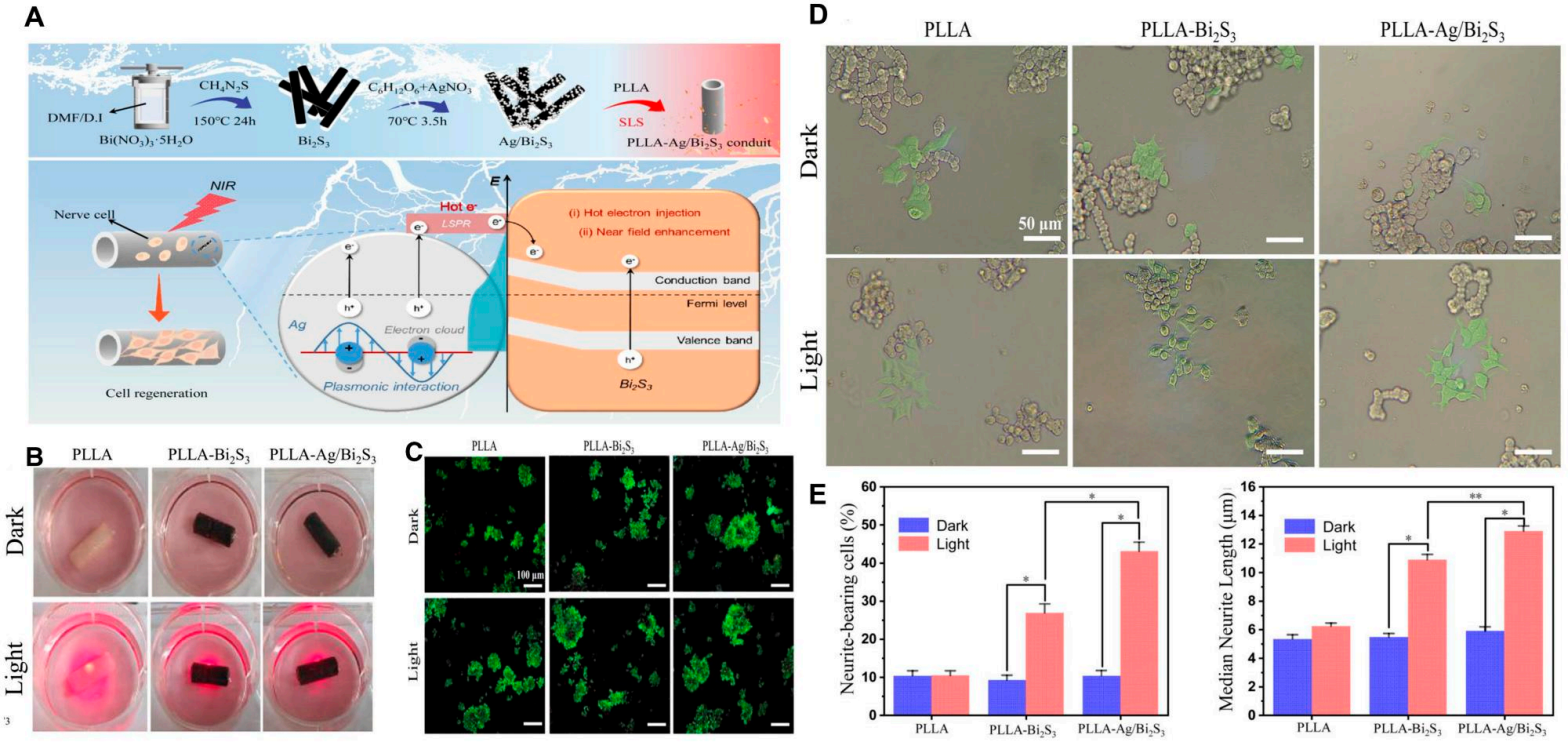
Photocurrent enhanced cell differentiation. (**A**) Diagrammatic representation of NGCs fabrication and use of PLLA-Ag/Bi_2_S_3_ for neural differentiation and nerve regeneration. (**B**) Representational image of conduit and cell co-culture and (**C**) cells stained by calcein-AM (green, live cells) and propidium iodide (red, dead cells) after with or without 808 nm NIR irradiation (**D**) images of PC12 cell differentiation on different substrates. (**E**) Percentages of differentiated PC12 and average neurite length of differentiated neurons. With approval, reprinted from Ref [573]. Copyright 2022, Elsevier.

Additionally, Qi and coworkers assessed the use of Ag/Bi_2_S_3_ with poly-L-lactic acid (PLLA) to fabricate a PLLA-Ag/Bi_2_S_3_ nerve guide conduit. Bi_2_S_3_ was to generate the photocurrent with the Ag NPs due to the localized surface plasmon resonance effect allowing for the effective redistribution of charge density under light irradiation and serving as electron mediators for the acceleration of electron transfer to boost the photocurrent (Figure 11A) [564–569]. The examination of PLLA-Ag/Bi_2_S_3_ under a cytotoxicity test showed negligible toxicity in PC12 cells even in the presence of light irradiation, which implied the provision of a conducive environment for the growth of cells (Figure 11B and C) [536, 570–572]. Similarly, on the differentiations of cells, PC12 cells showed high differentiations with long synapses, increased neurite length and high correlation of expression for the axonal growth associated with mRNA Nestin (Figure 11D and E). Which implicated that the photocurrent generated by the PLLA-Ag/Bi_2_S_3_ enhances the differentiation of nerve cells and the growth of synapses. Also, this was attributed to the Ca^2+^ which is involved in the signal transduction pathway, the modulation of neurotransmission, and synaptic plasticity. The conduit under NIR irradiation presented a Ca^2+^ influx due to the photo generation of electrons which triggered the voltage-gated calcium channel allowing the extracellular inflow of Ca^2+^. Further, the inflow of Ca^2+^ caused the intracellular signaling cascade to be activated which further upregulated SYNI protein, a neurogenesis-related protein. The function of the Ca^2+^ was just due to the normal functioning of the cells in the absence of any induced form of injury. This emphasizes the possibility of employing photo-electrical stimulation in the nerve generation process [536].

## Conclusion and the future outlook

Understanding the cellular and molecular activities involved during TON has the possibility of informing the use of the NGC since over a period of time a number of commercially produced NGC and conventional drugs have not been able to yield any spectacular results regarding the regeneration and functional recovery of the optic nerve. Firstly, the identification and distinguishing of resident microglia/infiltrating myeloid cells from the cluster of activated microglia and macrophage may seems to be imperative in the injured optic nerve and even at the progressive phase of ocular inflammation hence the need for the development of highly specific marker since they even function differently. The NGC may be rightly synthesized factoring all other qualities of a good scaffold or conduit however the vivid understanding involving the cellular activities seeks the chances of developing a scaffold that could function, mimic and present conditions appropriately required for the optic nerve regeneration and functional recovery. Studies regarding the use of NGC for the regenerating of the optic nerve over the years seem to be few. However, in the peripheral nerve, this has widely been employed especially in rodent studies with tremendous results. The issue with optic nerve regeneration will require a carefully fabricated scaffold taking into consideration, the toxicity, biocompatibility, and biodegradability. On the subject of toxicity, there are concerns about the delicate nature of the eye with its accessories and the development of secondary complications or even the risk of another ailment. Nonetheless, the fabrication process can consist of one or more biomaterials not limited to one to easily reduce the risk of toxicity, also another key determinant, contributing to toxicity is the materials used in the fabrication process. Since some of the materials are extremely toxic yet may be required for use depending on the approach or method one would want to use. Hence replacing them with environmentally friendly materials to yield similar effects without toxicity. With respect to biodegradability, for the successful use of a scaffold, there should be a possibility of its degrading without other surgical procedures to remove it, some polymers do have the chance of biodegrading within the cellular environment and also expelled at the same time without any secondary complications. Also, small sized nanomaterials may not have high stability yet are characterized by rapid degradation, such nanomaterials are highly required for scaffold fabrication. Additionally, the physicochemical state of biomaterials is employed, factoring the shape, size, thickness and other surface functionalizations. Normally highly porous scaffolds pave the way for the infiltration of inflammatory cells and growth factors diffusion. Conventionally, controlling such factors may seem quite challenging however the possible regulation has the tendency of enhancing the efficiency of the nanomaterials or the scaffold.

Although, the use of the NGC will still require some form of microsurgical process (direct end-to-end suturing), the use of autograft even in the peripheral nerve injury (PNI) has been quite challenging with accompanying pain, mismatch, and neuroma formation with increased cost. [466, 483, 573–577]. The orbit is a compartment housing delicate structures that are very crucial for vision and therefore surgical approaches require specialists (neurosurgeons and oculoplastic surgeons) to ensure the approach's, essential anatomical structure and function are safe [578–582]. Aside from the optic nerve, orbital injuries may require surgical treatment (orbital rim, zygomaticomaxillary, nasoorbitalethmoid, etc.). The complexity of the orbital anatomy points out the need for an appropriate surgical approach for exposure, optimization and successful treatment. In humans, the optic nerve happens to be located approximately 42 mm from the anterior lacrimal crest [583–586]. Therefore, its exposure happens to be the only way the NGC can be employed, modern radio-diagnostic technique easily allows for the site or location of the target. Over the years a number of approaches to orbitotomy have emerged since its description in 1889 (Kronlein) followed by its modification in 1953 (Berke), 1960 (Stallard-Wright), and 1976 (Maroon and Kennerdell) [587–590]. Although this approach has been broadly associated with orbital tumors located in proximity (dorsally, basally, laterally) to the optic nerve [591–594]. Hussein and coworkers detail how cuts and incisions are made at locations in proximity to the eyeball, outlining some crania orbital approaches such as the (frontotemporal approach, orbitozygomatic approach, supraorbital keyhole approach, Transorbital keyhole approach, etc.), an example in the use of CNTF-Chitosan, Liu Xiao and coworkers employed supratemporal intra-orbital approach in the rodent. However, as far as NGC is concerned employing endoscopic endonasal approaches might not be feasible although the approach can be used for delivery or also reaching the optic nerve [582, 586, 595]. The futuristic chances’ of fully employing the NGC in optic nerve regeneration seem not to be far away from the present research strive.
